# Identification and analysis of DNA-binding transcription factors in *Bacillus subtilis *and other Firmicutes- a genomic approach

**DOI:** 10.1186/1471-2164-7-147

**Published:** 2006-06-13

**Authors:** Samadhi Moreno-Campuzano, Sarath Chandra Janga, Ernesto Pérez-Rueda

**Affiliations:** 1 Departamento de Ingeniería Celular y Biocatálisis, Instituto de Biotecnología, Universidad Nacional Autónoma de México, Cuernavaca, Morelos, 62100, México; 2 Programa de Genómica Computacional, Centro de Ciencias Genómicas, Universidad Nacional Autónoma de México, Cuernavaca, Morelos, 62100, México

## Abstract

**Background:**

*Bacillus subtilis *is one of the best-characterized organisms in Gram-positive bacteria. It represents a paradigm of gene regulation in bacteria due its complex life style (which could involve a transition between stages as diverse as vegetative cell and spore formation). In order to gain insight into the organization and evolution of the *B. subtilis *regulatory network and to provide an alternative framework for further studies in bacteria, we identified and analyzed its repertoire of DNA-binding transcription factors in terms of their abundance, family distribution and regulated genes.

**Results:**

A collection of 237 DNA-binding Transcription Factors (TFs) was identified in *B. subtilis*, half of them with experimental evidence. 59% of them were predicted to be repressors, 17% activators, 17% were putatively identified as dual regulatory proteins and the remaining 6.3% could not be associated with a regulatory role. From this collection 56 TFs were found to be autoregulated, most of them negatively, though a significant proportion of positive feedback circuits were also identified. TFs were clustered into 51 regulatory protein families and then traced on 58 genomes from Firmicutes to detect their presence. From this analysis three families were found conserved in all the Firmicutes; fifteen families were distributed in all Firmicutes except in the phyla Mollicutes; two were constrained to Bacillales and finally two families were found to be specific to *B. subtilis*, due to their specie specific distribution. Repression seems to be the most common regulatory mechanism in Firmicutes due to the high proportion of repressors in the detected collection in these genomes. In addition, six global regulators were defined in *B. subtilis *based on the number and function of their regulated genes.

**Conclusion:**

In this work we identified and described the characteristics associated to the repertoire of DNA-binding TFs in *B. subtilis*. We also quantified their abundance, family distribution, and regulatory roles in the context of Firmicutes. This work should not only contribute to our understanding of the regulation of gene expression in bacteria from the perspective of *B. subtilis *but also provide us the basis for comprehensive modeling of transcriptional regulatory networks in Firmicutes.

## Background

Transcriptional regulation is an important mechanism for controlling many biological phenomena, such as development and cell proliferation. Regulation of gene expression in an organism involves a complex network mediated by DNA-binding transcription factors (TFs) which respond to changes in the cellular environment by altering the gene expression of relevant genes. Due to the crucial role of TFs in co-ordinating the gene expression kinetics of a genome, they are studied in many ways, including mutation analysis and elucidation of numerous three-dimensional structures. The identification of the repertoire of regulatory proteins in a genome sequence is a prerequisite to understand the regulation of gene expression and on a global scale for the elucidation of regulatory networks.

*B. subtilis *(GenBank: AL009126) is a sporulating Gram-positive bacterium that lives primarily in the soil and associated water sources. In its natural habitat, the bacterium is exposed to frequently changing environmental conditions. The high variability of the natural *B. subtilis *habitat is reflected in its complex gene regulatory apparatus enabling fast and efficient adaptation of the cell to varying environmental factors. Additionally, *B. subtilis *has evolved to develop a nearly inanimate physiological state, the spore [1]. Starvation and stress as well as initiation of spore formation (sporulation) and the further process of spore germination towards a vegetative cell are associated with extensive changes in the gene expression patterns. This results in a qualitative and quantitative variation in the composition of the cellular mRNA-pol [2].

*B. subtilis *is the best-characterized member of the Gram-positive bacteria. Its genome comprises of 4,224 protein-coding genes. Of these protein-coding genes, 53% are represented once, while a quarter of the genome corresponds to several gene families that have been greatly expanded by gene duplication, the largest family containing 77 putative ATP-binding transport proteins [3]. In addition, a large proportion of the genetic capacity is devoted to the utilization of a variety of carbon sources, including many plant-derived molecules. The publication of its genome sequence, subsequent systematic functional analysis and experimental characterization of its specific gene regulatory programs together with an extensive understanding of its biochemistry and physiology makes this micro-organism an excellent candidate next only to *Escherichia coli *K12 to model regulatory networks *in silico*.

In this work we are not only interested in the identification and classification of the DNA-binding transcriptional regulatory repertoire of *B. subtilis *but also in a comparative genomic analysis to deduce thereof how the TFs and their evolutionary families have been distributed among all Firmicutes sequenced. In the process we also characterize the TF repertoire in completely sequenced genomes of Firmicutes. We have selected this bacterium because it represents a model organism for Gram-positive bacteria, a group that includes a wide diversity of organisms, some of them pathogens and others important for the biotechnology industry. This analysis not only resulted in the identification of a core set of regulatory genes for *B. subtilis *and other organisms but also in the identification of a specific set involved in the regulation of gene expression in only this bacterium. This work provides a basis for the analysis of transcriptional regulatory networks in Firmicutes and beyond.

## Results and discussion

### The repertoire of DNA-binding TFs in *B. subtilis*

The identification and characterization of the repertoire of DNA-binding TFs in *B. subtilis *is a key step to understand the transcriptional gene regulatory machinery in this bacterium and opens an excellent opportunity to explore other Firmicutes. Therefore, we scanned the whole genome sequence of *B. subtilis *by using different computational approaches. Based on HMM-sequence comparisons and literature lookup, we identified a total of 237 genes as the minimal repertoire of proteins acting as DNA-binding regulators, that *B. subtilis *needs to regulate around 4200 genes (organized in approximately 2591 Transcription Units). 113 TFs have been experimentally characterized while 124 are predicted *in silico *(see Methods) [See additional file 1]. This dataset represents around 6.0% of the total protein coding genes of *B. subtilis *and is in agreement with previous estimates that suggest that less than 10% of gene products in all bacteria are associated to gene regulation [4]. However, alternative regulatory mechanisms such as sigma factors or attenuators which are excluded from this analysis could contribute to the regulatory complexity in this genome which otherwise suggests that a smaller proportion of the genome is devoted to transcription factors than in *E. coli *[4,5]. We found that the proportion of TFs to the number of Transcriptional Units (TUs) is 1:10, a similar proportion has been reported for *E. coli*. Additionally, based on the information detailing the regulation of 728 genes (promoters, TFs, and binding sites) deposited in the DBTBS [6], Subtilist [7], and Prodoric DB [8] we found that 61% of this set of genes in *B. subtilis *are regulated by one TF suggesting that gene regulation mediated by only one transcriptional regulator seems to be the most frequent case in most bacterial systems described so far [9]. 24% of the genes are regulated by two TFs, 11% are regulated by three TFs and around 5% by four or more TFs. However it should be noted that the data used in this analysis for the regulatory interactions of *B. subtilis *is far from complete and so the tendencies observed above although coherent with those observed in *E. coli *could be influenced by the incompleteness of the data in both the genomes. In the process of identifying regulators which have an influence on a large fraction of the regulatory network we defined a TF as a global regulator if it regulates more than 20 different genes organized on different transcription units (TU's) and if those genes belong to a minimum of four different functional categories, excluding hypothetical functions. This definition of identifying global regulators is based on previous observations made for the gram negative model, *E. coli *[10]. Using this approach, we identified six TFs (see Table 1) modulating the expression of around 60% of the total genes in *B. subtilis *as global regulators. These proteins regulate diverse processes including cellular mechanisms related to cellular envelope, amino acid biosynthesis, energy, and transport. Unlike this approach a recent work identified global regulators or hubs in the transcriptional regulatory network of *B. subtilis *using only the out-going connectivity of the TF [11], however the results suggest very good overlap. An intriguing observation is the case of the catabolic repression, mediated by the global regulator CcpA (Genbank: 16080026) in *B. subtilis *(Catabolite response regulator) which in *E. coli *is mediated by Crp (Genbank: 82583733), both proteins do not share similar evolutionary histories, but regulate the same metabolic response. Based on these two cases, one can infer that multiple mechanisms of catabolite control might have evolved independently of each other to respond to the same cellular condition, such as Crp (Crp family) in *E. coli*, CcpA (GalR/LacI family) in *B. subtilis*, and more recently Crc TF (Genbank: 15600525) (endonuclease/exonuclease/phosphatase family) in *Pseudomonas aeruginosa *[12].

**Figure 1:**
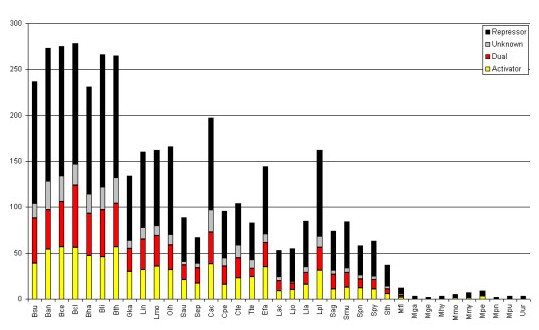
**Total number of TFs identified per genome for all the firmicutes analyzed in this study**. The proportion of activators (yellow), repressors (black), dual (red) and proteins with unknown (gray) regulatory role is shown.

**Figure 2:**
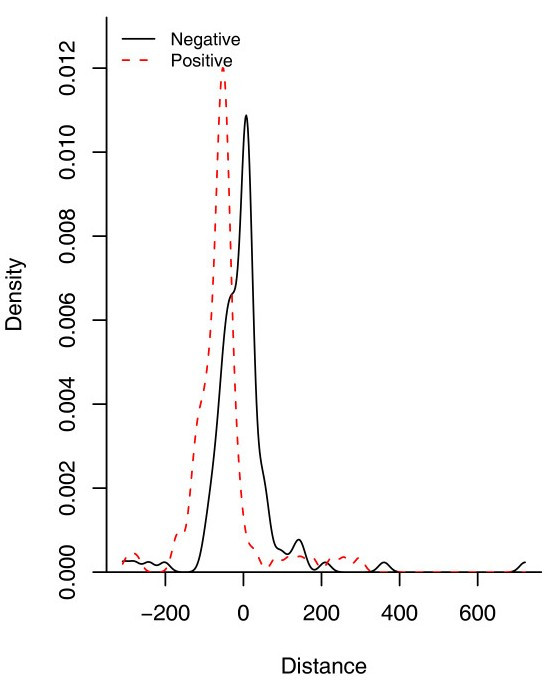
**Distribution of DNA-binding sites in *B. subtilis *for repressors and activators**. Data was retrieved from DBTBS [6]. 0 represents the start of transcription site (+1). The data was fitted using a Gaussian kernelwith a bandwidth of 13.92 for repressor sites and12.42 for activator sites using the density estimation function in the R statistics package.

In bacteria, the most common structure associated to TFs is the helix-turn-helix (HTH). The position of this structure in the sequence correlates with the regulatory role, i.e. most repressors tend to have a HTH in the N-terminal whereas the activators display it in the C-terminal [13]. In order to determine the proportion of repressors, activators and dual TFs in *B. subtilis*, we used this correlation to assign probable regulatory roles to the collection of regulators in the dataset where the DNA-binding structure is a HTH, some of them were corroborated by literature search. From this analysis, we found that 59% of the TFs could be predicted to be repressor proteins, 17% activators and 17% could be putatively assigned as dual regulators while for 6% of them a regulatory role could not be deduced (See Figure 1). This trend correlates with the observation that most promoters are repressed in bacteria and correlates with the fact that *B. subtilis *exhibits a major proportion of promoters repressed than activated. Indeed, a more detailed analysis of these promoters indicates that around 60% of the repressor sites are between -1 to -60, the region occupied by the RNA pol, whereas around 30% are between +1 to +60 (see Figure 2), suggesting that repression by steric hindrance is probably the most common regulatory mechanism associated with the TFs in *B. subtilis*, where the repressor-binding site overlaps core promoter elements and blocks recognition of the promoter by the RNA polymerase holoenzyme [14], whereas in lesser proportion by blocking the elongation chain. On the other hand, 60% of the positive sites were found to be located upstream of the promoter (between -40 to -100) suggesting a mechanism of activation of class I and II, where the activator binds to a target that is adjacent to the promoter's -35 element, and the bound activator interacts with the alpha subunit of the region 4 of σ^70^. 15% of the activated DNA-binding sites are located between -40 to -1 suggesting activation by conformation changes, where the activator binds at or near to the promoter elements and realigns the -10 element and the -35 element so that the RNA polymerase holoenzyme can bind to the promoter [14]. Similar trends have been observed in two independent studies conducted previously using the data from *E. coli *[15,16].

**Figure 3:**
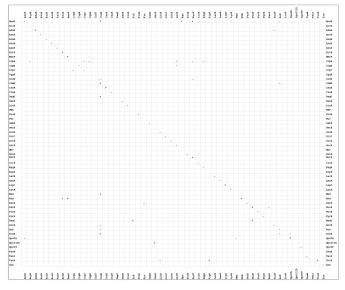
**Matrix of characterized interactions among TFs in *B. subtilis***. Each filled box shows the sign of the regulatory effect of the TF in the corresponding column on the expression of the TF in the corresponding row. Repression is denoted by -, activation is denoted by + and dual regulation is denoted by *.

Additionally we identified 56 TFs, which are reported to be cross-regulated. In Figure 3 we show the matrix of regulatory interactions for these TFs in *B. subtilis*. From this dataset 69% were found to be negatively, 26% positively and 5% dual autoregulated among those whose auto-regulatory role could be established. A similar trend has been observed previously in the case of *E. coli *K12 [4]. However, the proportion of auto-regulatory positive feedback circuits in *B. subtilis *contrasts against that observed in *E. coli*, where only 6.5% account for positive autoregulation. Probably, this difference is a consequence of the enhanced regulatory mechanisms in *B. subtilis *which could have been developed for a systematic recruitment of metabolic signals to improve the response or to switch between vegetative and spore life cycle. In fact, Thieffry et. al [17] propose that mixed metabolic/genetic positive circuits need the continuous presence of the involved metabolites to remain active, allowing the cell to monitor the presence of such metabolites continuously. In light of these findings we propose that the common autoregulatory organization observed in the TFs of *E. coli *and *B. subtilis*, might play an important evolutionary and functional role in all bacteria, due to the fact that perturbations in the expression of a particular TF would lead to a change of expression of a limited number of coordinated genes, and not to the whole network.

### Identification of TF families in *B. subtilis*

In a previous analysis it has been proposed that DNA-binding TFs can be grouped into protein families based on their amino acid sequence similarity [4]. In order to construct TF families in *B. subtilis*, we first identified and defined families based on HMM searches done with family specific HMMs derived in *E. coli *and by using PFAMs [18] (see Methods). In order to expand the families we then considered a protein as a member of a family if it shared at least 25% of identity with any member of the group in the DNA-binding domain (DBD) or if the protein had matches derived from HMM searches. We then performed alignments between the TF and its corresponding family-specific PFAM model, by using the hmmalign program from HMMer suite of programs [18].

**Figure 4:**
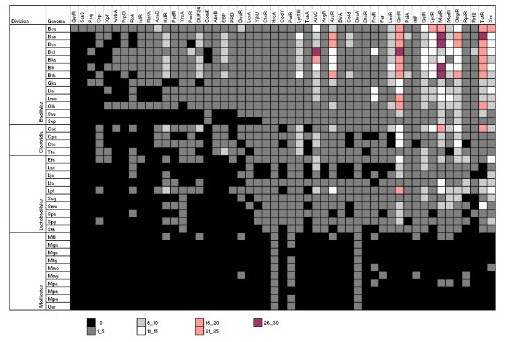
**Distribution of TF families and their abundance in Firmicutes**. We present the abundance of TFs per genome at intervals of five proteins. Nomenclature is as follows: Bacillales: Ban, *Bacillus anthracis *Ames Ancestor; Bce, *B. cereus *10987; Bcl, *B. clausii *KSM-K16; Bha, *B. halodurans *C-125; Bli, *B. licheniformis *ATCC14580 (DSM 13); Bsu, *B. subtilis *168; Bth, *B. thuringiensis konkukian*; Gka, *Geobacillus *kaustophilus HTA426; Lin, *Listeria innocua *CLIP 11262; Lmo, *L. monocytogenes *4b F2365; Oih, *O. iheyensis *HTE831; Sau, *Staphylococcus aureus *subsp. aureus COL; Sep, *S. epidermidis *ATCC 12228. Clostridia: Cac, *Clostridium acetobutylicum *ATCC824; Cpe, *C. perfringens *13; Cte, *C.tetani *E88; Tte, *Thermoanaerobacter tengcongensis *MB4(T). Lactobacillales: Efa, *Enterococcus faecalis *V583; Lac, *Lactobacillus acidophilus *NCFM; Ljo, *L. johnsonii *NCC 533; Lla, *Lactococcus lactis *subsp. lactis IL1403; Lpl, *L. plantarum *WCFS1; Sag, *Streptococcus agalactiae *2603V/R; Smu, *S. mutans *UA159; Spn, *S.pneumoniae *TIGR4; Spy, *S.pyogenes *MGAS315; Sth, *S.thermophilus *CNRZ1066. Mollicutes: Mfl, *Mesoplasma florum *L1; Mhy, *Mycoplasma hyopneumoniae *232; Mmo, *M. mobile *163K; Mmy, *M.mycoides *SC PG1; Mga, *M.gallisepticum *strain R; Mge, *M.genitalium *G-37; Mpe, *M.penetrans *HF-2; Mpn, *M.pneumoniae *M129; Mpu, *M.pulmonis *UAB CTIP; Uur, *Ureaplasma urealyticum *parvum biovar serovar 3.

The whole repertoire of TFs was clustered into 51 families of varying sizes (see Figure 4), for instance, nine families contain more than 10 members, the most abundant being the Multiple Antibiotic Resistance Regulators (MarR) family (20 TFs) and the GntR family (14 TFs); whereas thirty-nine families include less than 9 TFs. An interesting observation is that the family ArsR (Arsenic resistance regulator) contains nine members, while in *E. coli *this family is represented by only two proteins, suggesting diverse events of gene duplication for members of the family in this bacterium. Another notable difference relative to *E. coli *is the Crp family: in *E. coli *three members associated to global regulatory processes have been identified, while in *B. subtilis *only Fnr was identified. This observation suggests the possibility that Fnr could have existed in the last common ancestor of these two genomes and that gene loss could have been responsible for the absence of other members, however additional evidences are necessary. Twenty-six genes encoding response regulators were also identified (from the family of EBP, LuxR and OmpR), most of which are arranged adjacent to genes encoding histidine kinases. This finding is interesting because it represents a probable co-evolution process between the response regulators and sensor genes. Indeed, recent co-evolution events have been identified in members of this family in *E. coli *[19].

In addition, 19 families include only one member per group. These families seem to play an important role in specific processes of this cellular division, such as sporulation, and bacterial competence, such as AbrB, ComK, and CodY families. Local regulators, such as BirA (biotin biosynthesis), LexA (SOS response), Fur (Ferric uptake regulator) or ArgR (arginine biosynthesis regulation) families were also identified in few copies. A similar trend has been found in different bacterial genomes for these TFs [20].

In summary, we found a smaller proportion of families in *B. subtilis *in comparison to *E. coli *K12. This difference is more remarkable when we see the number of members per family, in *E. coli *the LysR family is the largest (up 45 TFs), while in *B. subtilis *MarR is the largest family identified so far (20 TFs). These families are associated to different physiological functions (LysR, amino acid biosynthesis and MarR resistance to antibiotics). We also found that there are specific regulators associated to *B. subtilis *and to Firmicutes, three of them were involved in sporulation and related processes (AbrB, ComK, and CodY). This difference, in the proportion of TFs and families, suggest that different regulatory mechanisms have been probably invented in *B. subtilis *to specific processes, such as sporulation, but also sharing a core of TFs to maintain an adequate homeostatic control in the rest of the genes.

### Structural assignments and Fold frequency of Transcription factors (TFs)

Helix-Turn-Helix (HTH) is known to be the most common structure associated to DNA-binding TFs in bacteria [5,20]. Alternative structures have been identified in smaller proportions. In order to determine the diversity of the TF structural domains in the repertoire of TFs in *B. subtilis*, the transcriptional regulators were analysed by using Superfamily HMM models [21]. This analysis shows the structural variability associated to HTH proteins. We found that fourty-seven percent of the whole repertoire of TFs contain a "winged" HTH. This result is interesting because it represents 21 out of 51 families identified in this bacterium. Around 14% of the TFs (that represent four families) are intimately associated to the "homeodomain-like" HTH superfamily domain. Only two families contained the "classical" HTH, although representing almost 12% of the whole repertoire, showing that these groups represent two largest families in *B. subtilis *(GalR/LacI and Xre).

Alternative DNA-binding domains were also identified, though in much smaller proportions, such as the IHF-like structures or nucleic acid binding structures (associated to the Cold Shock family). Finally, *B. subtilis *TFs contain two-domain proteins (a DBD and a multimerization/ligand binding domain). A similar trend has been noticed in the repertoire of TFs in *E. coli K12 *reported recently [22], where the authors also suggest that almost three quarters of the TFs are two-domain (like in *B. subtilis*), and are a result of diverse duplication events [See additional files 2 and 3].

### Tracing the TF families in Firmicutes

In order to determine the abundance and distribution of TF families among Firmicutes, we examined their occurrence in 58 genomes, 27 Bacillales, 17 Lactobacillales, 10 Mollicutes, and 4 Clostridia (see Methods). We considered this analysis under the belief that it might give us clues about the evolution of common cellular processes among organisms of this bacterial lineage. Below we summarize the prominent observations emerged out of this analysis:

a) We observed a rough trend between the numbers of TFs versus the genome size. Larger genomes contain more TFs than smaller ones. This might not be a surprise considering that the more number of coding regions within a genome it would encode more DNA-binding transcriptional regulators, like it has been previously proposed [5,20,23,24]. Thus, the proportion of TFs in larger genomes would be consistent with the hypothesis that an increase of genome complexity and physiological functionality is generally associated with a more complex regulation of gene expression since the additional genetic information has to be integrated into the existing regulatory networks that operate in a bacterial cell [25]. In this context it is interesting to note that the phyla Molliscutes contains a much smaller fraction of TFs identified so far, probably because most of these organisms have lost a lot of their genes as a consequence of their life style (See below and Figure 4).

b) When the proportion of TFs was analyzed as a function of number of families *per *genome, we found that although some bacteria contain a high proportion of families, their sizes seem to be reduced, whereas in bacteria with few families the familial sizes seem to be larger with high proportion of TFs. This finding suggests that some families have been widely duplicated, whereas other families have been constrained to few members as a consequence of the bacterial life style. This could also suggest that a fraction of the total TF repertoire in Firmicutes is a consequence of massive gene duplication constrained to only few protein families. For instance, whereas *B. licheniformis DSM 13 *is the bacteria with the largest repertoire of TFs (278 TFs and 46 families) it contains the same number of families as *Oceanobacillus iheyensis *(166 TFs and 46 families) or *Geobacillus kaustophilus *HTA426 (134 TFs and 43 families).

c) We identified three families "universally" distributed among Firmicutes which include HrcA, DnaA, and PurR (except in the mollicutes *Mycoplasma genitalum *and *M. pneumoniae*). These families are associated to the regulation of class I heat-shock genes (*dnaK*, *groESL*) for HrcA, DNA replication process (DnaA), and the adenine nucleotide-dependent regulation of *pur *operon for PurR; all of them important informational processes and they might belong to the ancestral core of TFs in this cellular division.

d) Fifteen families were identified as common families to Bacillales, Lactobacillales and Clostridia, which include GntR, GalR/LacI, LysR, MarR, TetR, MerR, OmpR, RpiR, Rrf2, CtsR, LytR, AraC/XylS, Xre, IHF and PaiA. Interestingly, many of these families are highly represented in these three lineages of Firmicutes. Members of the GntR, AraC/XylS and GalR/LacI families generally respond to environmental changes that affect the carbohydrate metabolism of the cell [4]. It certainly makes sense that soil bacteria have a large diversity of DNA-binding transcriptional regulators that respond to changes in the carbohydrate composition of the environment. The families MarR, TetR and MerR regulate the resistance to antibiotics and mercury among others, while the family OmpR is associated to regulate membrane components from the two component systems. The large number of proteins grouped into GntR and GalR/LacI families may provide these bacteria the ability to grow in the presence of several carbon sources and to rapidly adapt their gene expression to changing nutrient conditions as has been suggested previously [26].

e) Two families exclusive to Bacillales: Psq and ComK were traced among all genomes of Bacillales. Among these ComK, emerges like an essential TF for the development of genetic competence in *B. subtilis *and probably in all Bacillales. This protein contains an atypical DNA-binding structure, the "helical domain of sec23/24" [27]. This transcription factor is considered as a global regulator and its gene expression is strictly regulated by nutritional and growth phase- dependent control [28]. Additionally, it is dependent on its own gene product and that of the TFs AbrB, ComA, ComP, DegU, Sin, Spo0A, Spo0H, Spo0K and SrfA. This system is highly regulated because it represents the final convergence signals which trigger competence development [29]. The highly regulated genes might be associated to key processes in the cell, such as competence or sporulation suggesting that additional genes highly regulated might participate in important cellular mechanisms. It should be noted that most Bacillales include a phase of sporulation in their life cycle.

f) Finally, two families, GutR and SenS, were exclusively identified in *B. subtilis*. These families are very interesting as they seem to be intimately related with important cellular processes, such as the regulation of sorbitol dehydrogenase gene (*gutB*) by GutR, which contains a HTH motif and a nucleotide binding domain at the N-terminal region [30] and regulation of extracellular enzyme genes (*amyE*, *aprE*, *nprE*) by SenS, which comprises of a HTH motif along its length of 65 amino acids [31-33]. These TF families might be considered as a regulatory signature of this bacterium.

In summary, the distribution and abundance of TF families was traced among fifty-eight genomes of Firmicutes from different lineages, opening diverse opportunities to understand the evolution of regulatory networks in this bacterial division and to define their precise role in maintaining an adequate control of gene expression. This repertoire of TF families will also pave the way to understand and analyze exhaustively other Firmicutes from the perspective of *B. subtilis *and to consider other specific and important questions not addressed here.

## Conclusion

Based on analysis of the sequenced genome of *B. subtilis *we identified its repertoire of DNA-binding TFs, which allowed us to identify TFs common to other Firmicutes, and TFs specific to few closer lineages. We demonstrated that the number of TFs reflects different forms of life styles, and that families are distributed almost homogeneously among all Firmicutes. The diverse elements involved in the regulatory networks apparently have different evolutionary histories some times denoting exclusive functional conservation in specific lineages such as sporulation specific TFs observed in Bacillales. Further research is necessary to determine the physiological function of species-specific and shared transcriptional regulators that might be involved either in the regulation of cellular processes relevant for biotechnological production or that might control the expression of genes involved, for instance, in virulence of pathogenic bacteria. However, we must consider alternative regulatory mechanisms not considered here, such as attenuation or regulation mediated by riboswitches. For instance, when we analyzed the proportion of sigma factors between *E. coli *(7 sigma factors) and *B. subtilis *(17 sigma factors) we found a clear difference between them possibly suggesting that sigma factors account for the relatively large proportion of DNA-binding proteins in *B. subtilis *in comparison to *E. coli*. The analysis presented here, will help to understand the regulatory networks in different bacteria by using *E. coli *and *B. subtilis *as models and to decipher the evolution of these networks in a global context.

## Methods

### Identification of DNA-binding transcription factors (TFs)

In order to identify the repertoire of TFs in Firmicutes including *B. subtilis*, we used a combination of information sources and bioinformatics tools. The first set of 292 putative TFs were collected from DBTBS, a database devoted to the gene regulatory mechanism in *B. subtilis *strain 168 [6]. From this dataset, we excluded by sequence comparison against SwissProt and reference searches, 75 proteins annotated as terminators, antiterminators, and sigma factors, among others. Finally, we were left with 217 well-annotated TFs in this bacterium.

In the second phase, 90 family-specific Hidden Markov Models (HMMs) reported previously [20] were used to scan the whole *B. subtilis *sequence genome (E-value threshold ≥ 10-3). We used the hmmsearch module from HMMer suite of programs [18]. Briefly, these models were constructed by using as seed the TF families previously identified in *E. coli K12*. The models – almost exclusively- consider the DNA-binding domain sequence for every protein family (around 60 amino acids). We excluded proteins that matched less than 50% against their corresponding HMM although they correspond to the DBD. In this search, 181 proteins were identified as probable TFs [See additional file 4] This search is important because proteins identified by these specific HMMs might not be included in the dataset retrieved from the previous phase.

In the third phase, the *B. subtilis *proteome was analyzed with the library of HMMs from the Superfamily database (E-value ≥ 10-3) [21]. This HMM library is based on the sequences of domains collected in the Structural Classification of Proteins (SCOP) database [27] and are thus applicable for a structural classification of proteins. This attempt was made to identify additional TFs not identified in the previous phases.

TFs identified in each of the three phases: DBTBS, HMM-*E. coli *models and Superfamily searches were compared to define the final TF repertoire. The final dataset included the intersection of proteins identified by HMMs, Superfamily searches, and the repertoire (manually curated) of TFs described in DBTBS. Three confidence levels were considered to have an assessment of the quality of the identified TFs: a) higher level that includes TF identified by the three approaches; b) medium level, those identified by two methods; and c) lower level, for TFs which are identified by only one method. Additionally, literature information was used to find additional TFs not identified by these automatic searches.

Finally, 237 proteins were deduced like the minimal number of TFs that *B. subtilis *needs to regulate its gene expression. The identified proteins were classified into families by using HMMs deposited in the PFAM DB [18], and aligned by using the program hmmalign from HMMer. In order to identify TFs and families in other Firmicutes we constructed family-specific HMMs to *B. subtilis*, and we ran against 58 Firmicute genome sequences (E-value ≥ 10-3 was considered as threshold) [See additional file 5].

## Abbreviations

TF, Transcription Factors

DBD, DNA-Binding Domain

HMMs, Hidden Markov Models

SCOP, Structural Classification of Proteins

HTH, Helix-Turn-Helix

TUs, Transcriptional Units

Crp, CcpA, Crc, Catabolite response regulators

σ, Sigma factor

## Authors' contributions

S. M-C carried out the compilation of DNA-binding TFs from diverse DBs. E. P-R participated in the comparative genome analyses and drafted the manuscript. SC.J participated in data analysis and helped to draft the manuscript. All authors read and approved the final manuscript.

## Supplementary Material

Additional File 1 Table S1. Collection of 237 DNA-binding TFs identified in *B. subtilis*. Columns are as follows: Common name, Protein ID, family, BG_ID, confidence level (identification method) and regulatory role. Click here for file

Additional File 2 Table S2. Functional description of regulatory families. Columns are as follows: Family name, number of members, regulatory role, HTH position, DNA-binding structure and physiological role. Click here for file

Additional File 3 Table S3. Identification of TFs by Superfamily searches. Nomenclature: Gene ID, Ids from Superfamily (DBD), and Domain Position. Click here for file

Additional File 4 Table S4. Identification of TFs by HMM family specific. Columns are as follow: Gene ID, Family name, start and end position domains in the TF, bit and E-value scores. Click here for file

Additional File 5 Table S5. Genomes analyzed in this work. Columns are as follows: Cellular division, order, Organism name, Taxonomic Identifier, Genome size (Mbp), number of TFs and Genome code used in the manuscript. Click here for file

## Figures and Tables

**Table 1:** Global regulators. The numbers in braces denote the percentage of genes in the category regulated by the TF. In bold are the functional categories, which are dominantly regulated by the respective TF. Functional categories are as follows: AA, Amino acid biosynthesis; CPRO, Cellular processes; CEN, Cell envelope; CIM, Central intermediary metabolism; DNA, DNA metabolism; ENER, Energy metabolism; FATE, Protein fate; MOB, Mobile and extrachromosomal element functions; Signal, Signal transduction; PUR, Purines, pyrimidines, nucleosides and nucleotides; REG; Regulatory functions; Transport, Transport and binding proteins.

**Protein**	**Functional Category**	**Regulatory role**	**Family**
AbrB	AA (10.2); CPRO (16.3); REG (30.6); Transport (18.3)	Regulates the transcription of genes expressed during the transition state between vegetative growth and the onset of stationary phase and sporulation	AbrB
CcpA	CPRO (6.6); ENER (33.9); REG (13.2); Signal (4.7); Transport (26.4)	Repression of the carbohydrate utilization genes; and in the positive regulation of genes involved in excretion of excess carbon.	LacI
CodY	AA (22.5); MOB (12.5); REG (20.0); Transport (12.5)	Repression of genes induced as cells make the transition from rapid exponential growth to stationary phase and sporulation.	CodY
ComK	AA (12.1); CEN (15.6); CPRO (13.9); DNA (7.8); ENER (7.8); FATE (6.0); PUR (7.8); REG (10.4); Transport (6.9).	Intermediate regulatory gene required for the expression of the late competence genes.	ComK
SpoOA	CPRO (17.0); PUR (23.1); REG (18.2); Transport (7.3)	Initiation of sporulation (negative regulation of abrB, kinA, kinC, spo0A; positive regulation of spoIIA, spoIIE, spoIIG)	OmpR
TnrA	CIM (18.5); ENER (18.5); Transport (25.9)	Regulates genes during nitrogen-limited growth	MerR
