# Supplementary material for: Identification and analysis of DNA-binding transcription factors in Bacillus subtilis and other Firmicutes- a genomic approach
Source: BMC Genomics. 2006 Jun 13;7:147. doi: 10.1186/1471-2164-7-147 (PMC1524751; doi:10.1186/1471-2164-7-147)
Supplement: Additional File 1 — Table S1. Collection of 237 DNA-binding TFs identified in B. subtilis. Columns are as follows: Common name, Protein ID, family, BG_ID, confidence level (identification method) and regulatory role. [file 1471-2164-7-147-S1.doc]

| **Name** | **ID** | **Family** | **BG_ID** | **Confidence level** | **DB search** | **Superfamily** | **Family-specific** | **Role** |
| --- | --- | --- | --- | --- | --- | --- | --- | --- |
| Abh | 16078512 | AbrB | BG10988 | Medium | DBTBS | SUPFAM |  | **Dual** |
| AbrB | 16077105 | AbrB | BG10100 | Medium | DBTBS | SUPFAM |  | ***Dual*** |
| AcoR | 16077877 | Ebp | BG11790 | High | DBTBS | SUPFAM | HMM-*ad hoc* | **Activator** |
| AdaA | 16077249 | AraC | BG10166 | Medium | DBTBS |  | HMM-*ad hoc* | **Dual** |
| AhrC | 16079481 | ArgR | BG10309 | High | DBTBS | SUPFAM | HMM-*ad hoc* | **Dual** |
| AlsR | 16080655 | LysR | BG10470 | High | DBTBS | SUPFAM | HMM-*ad hoc* | **Dual** |
| AnsR | 16079416 | Xre | BG10299 | Medium | DBTBS | SUPFAM |  | **Repressor** |
| AraR | 16080450 | GntR | BG11913 | High | DBTBS | SUPFAM | HMM-*ad hoc* | **Repressor** |
| ArsR (yqcJ) | 16079634 | ArsR | BG11301 | High | DBTBS | SUPFAM | HMM-*ad hoc* | **Repressor** |
| AzlB | 16079725 | AsnC | BG11914 | High | DBTBS | SUPFAM | HMM-*ad hoc* | **Repressor** |
| BirA | 16079301 | BirA | BG11206 | Medium | DBTBS |  | HMM-*ad hoc* | **Repressor** |
| BkdR | 16079466 | Ebp | BG11721 | High | DBTBS | SUPFAM | HMM-*ad hoc* | **Activator** |
| BltR | 16079711 | MerR | BG10904 | High | DBTBS | SUPFAM | HMM-*ad hoc* | **Activator** |
| BmrR | 16079458 | MerR | BG10304 | Medium |  | SUPFAM | HMM-*ad hoc* | **Activator** |
| CcpA | 16080026 | LacI | BG10376 | High | DBTBS | SUPFAM | HMM-*ad hoc* | **Repressor** |
| CcpB (YyaG) | 16081139 | LacI | BG10045 | Medium |  | SUPFAM | HMM-*ad hoc* | **Repressor** |
| CcpC (YkuM) | 16078478 | LysR | BG13297 | High | DBTBS | SUPFAM | HMM-*ad hoc* | **Repressor** |
| CggR | 16080448 | DeoR | BG14085 | Low |  |  | HMM-*ad hoc* | **Repressor** |
| CitR | 16078008 | LysR | BG10853 | High | DBTBS | SUPFAM | HMM-*ad hoc* | ***Repressor*** |
| CitT | 16077826 | OmpR | BG12577 | Medium | DBTBS | SUPFAM |  | ***Activator*** |
| CodY | 16078680 | CodY | BG10968 | Medium | DBTBS | SUPFAM |  | ***Repressor*** |
| ComA | 16080219 | GerE | BG10381 | High | DBTBS | SUPFAM | HMM-*ad hoc* | ***Activator*** |
| ComK | 16078106 | ComK | BG11059 | Medium | DBTBS | SUPFAM |  | ***Dual*** |
| CspB | 16077975 | Cold-Shock | BG10824 | High | DBTBS | SUPFAM | HMM-*ad hoc* | ***Activator*** |
| CspC | 16077579 | Cold-Shock | BG11024 | Medium |  | SUPFAM | HMM-*ad hoc* | ***Activator*** |
| cspD | 16079252 | Cold-Shock | BG11531 | Medium |  | SUPFAM | HMM-*ad hoc* | ***Activator*** |
| CssR | 16080354 | OmpR | BG14131 | High | DBTBS | SUPFAM | HMM-*ad hoc* | **Activator** |
| CtsR | 16077151 | CtsR | BG10145 | Medium | DBTBS | SUPFAM |  | **Repressor** |
| DctR (YdbG) | 16077513 | OmpR | BG12074 | Medium | DBTBS | SUPFAM |  | **Activator** |
| DegA | 16078147 | LacI | BG10847 | High | DBTBS | SUPFAM | HMM-*ad hoc* | **Activator** |
| DegU | 16080602 | GerE | BG10393 | High | DBTBS | SUPFAM | HMM-*ad hoc* | **Activator** |
| DeoR | 16080994 | YjhU_YdeW | BG10982 | High | DBTBS | SUPFAM | HMM-*ad hoc* | **Repressor** |
| DesR (YocG) | 16078980 | GerE | BG13520 | High | DBTBS | SUPFAM | HMM-*ad hoc* | **Activator** |
| DinR | 16078848 | LexA | BG10678 | High | DBTBS | SUPFAM | HMM-*ad hoc* | **Repressor** |
| DnaA | 16077069 | DnaA | BG10065 | Medium | DBTBS |  | HMM-*ad hoc* | **Repressor** |
| ExuR (YjmH) | 16078302 | LacI | BG13211 | High | DBTBS | SUPFAM | HMM-*ad hoc* | Repressor |
| Fnr | 16080784 | Crp | BG11343 | Medium | DBTBS | SUPFAM |  | **Activator** |
| FruR | 16078502 | DeoR | BG12589 | High | DBTBS | SUPFAM | HMM-*ad hoc* | **Repressor** |
| Fur (YqkL) | 16079409 | FUR | BG11766 | Medium | DBTBS |  | HMM-*ad hoc* | **Repressor** |
| GabR (YcnF) | 16077457 | GntR | BG12042 | High | DBTBS | SUPFAM | HMM-*ad hoc* | **Dual** |
| GerE | 16079893 | GerE | BG10355 | High | DBTBS | SUPFAM | HMM-*ad hoc* | **Repressor** |
| GlcK | 16079541 | ROK | BG11685 | Medium | DBTBS |  | HMM-*ad hoc* | ***Repressor*** |
| GlcR | 16080683 | DeoR | BG12503 | High | DBTBS | SUPFAM | HMM-*ad hoc* | **Repressor** |
| GlnR | 16078808 | MerR | BG10424 | High | DBTBS | SUPFAM | HMM-*ad hoc* | **Repressor** |
| GltC | 16078907 | LysR | BG10810 | High | DBTBS | SUPFAM | HMM-*ad hoc* | **Dual** |
| GltR | 16079720 | LysR | BG11942 | High | DBTBS | SUPFAM | HMM-*ad hoc* | **Repressor** |
| GlvR | 2633143 | RpiR | BG11847 | Low | DBTBS |  |  | **Activator** |
| GntR | 16081057 | GntR | BG10648 | High | DBTBS | SUPFAM | HMM-*ad hoc* | **Repressor** |
| GutR | 16077681 | GutR | BG10178 | Medium | DBTBS | SUPFAM |  | **Activator** |
| HBS | 16079336 | IHF | BG10276 | Medium | DBTBS |  | HMM-*ad hoc* | **Unknown** |
| Hpr | 16078063 | MarR | BG10659 | Medium | DBTBS | SUPFAM |  | **Repressor** |
| HrcA | 16079603 | HrcA | BG10662 | Medium | DBTBS |  | HMM-*ad hoc* | **Repressor** |
| HxlR | 16077416 | HxlR | BG11184 | Medium | DBTBS | SUPFAM |  | **Activator** |
| IolR | 16081028 | DeoR | BG11364 | High | DBTBS | SUPFAM | HMM-*ad hoc* | **Repressor** |
| KdgR | 16079270 | LacI | BG11398 | High | DBTBS | SUPFAM | HMM-*ad hoc* | **Repressor** |
| KipR (YcsO) | 16077477 | IclR | BG11214 | High | DBTBS | SUPFAM | HMM-*ad hoc* | **Repressor** |
| LacR | 16080470 | LacI | BG12435 | High | DBTBS | SUPFAM | HMM-*ad hoc* | **Repressor** |
| LevR | 16079762 | PRD | BG10677 | Medium | DBTBS |  | HMM-*ad hoc* | **Activator** |
| LicR | 16080911 | FrvR | BG11346 | Medium | DBTBS |  | HMM-*ad hoc* | **Activator** |
| LmrA | 16077337 | TetR | BG12612 | High | DBTBS | SUPFAM | HMM-*ad hoc* | **Repressor** |
| LrpA | 16077572 | AsnC | BG12122 | High | DBTBS | SUPFAM | HMM-*ad hoc* | **Repressor** |
| LrpB | 16077573 | AsnC | BG12123 | High | DBTBS | SUPFAM | HMM-*ad hoc* | **Repressor** |
| LrpC | 16077492 | AsnC | BG12056 | High | DBTBS | SUPFAM | HMM-*ad hoc* | **Dual** |
| LytT | 16079944 | LytTR | BG11953 | Medium | DBTBS | SUPFAM |  | **Repressor** |
| MmgE | 16079469 | PrpD | BG11323 | Low |  |  | HMM-*ad hoc* | **Unknown** |
| MntR | 16079508 | DtxR | BG11702 | Medium | DBTBS | SUPFAM |  | **Dual** |
| MsmR | 16080078 | LacI | BG12626 | High | DBTBS | SUPFAM | HMM-*ad hoc* | **Repressor** |
| Mta | 16080713 | MerR | BG12482 | High | DBTBS | SUPFAM | HMM-*ad hoc* | **Activator** |
| OhrR (YkmA) | 16078380 | MarR | BG13239 | High | DBTBS | SUPFAM | HMM-*ad hoc* | **Repressor** |
| PadR | 16077901 | PadR | BG12220 | Medium | DBTBS |  | HMM-*ad hoc* | **Repressor** |
| PaiA | 16080268 | PaiA | BG10695 | Medium | DBTBS |  | HMM-*ad hoc* | **Repressor** |
| PerR (YgaG) | 16077938 | FUR | BG12227 | High | DBTBS | SUPFAM | HMM-*ad hoc* | **Repressor** |
| PhoP | 16079963 | OmpR | BG10363 | High | DBTBS | SUPFAM | HMM-*ad hoc* | **Dual** |
| PksA | 16078770 | TetR | BG12647 | Medium | DBTBS | SUPFAM |  | **Repressor** |
| PucR | 16080295 | PucR | BG13983 | Medium | DBTBS |  | HMM-*ad hoc* | **Dual** |
| PurR | 16077115 | PurR | BG10110 | Medium | DBTBS |  | HMM-*ad hoc* | **Repressor** |
| RbsR | 16080644 | LacI | BG10876 | High | DBTBS | SUPFAM | HMM-*ad hoc* | **Repressor** |
| ResD | 16079369 | OmpR | BG10534 | High | DBTBS | SUPFAM | HMM-*ad hoc* | **Activator** |
| RocR | 16081087 | Ebp | BG10723 | High | DBTBS | SUPFAM | HMM-*ad hoc* | **Dual** |
| Rok | 16078488 | Rok | BG13307 | Medium | DBTBS | SUPFAM |  | **Repressor** |
| RsfA | 16080814 | RsfA | BG10638 | Low |  |  | HMM-*ad hoc* | **Dual** |
| SenS | 16077946 | SenS | BG10747 | Medium |  | SUPFAM | HMM-*ad hoc* | **unknown** |
| SinR | 16079517 | Xre | BG10754 | Medium | DBTBS | SUPFAM |  | **Dual** |
| Slr | 16080491 | Xre | BG11858 | Medium | DBTBS | SUPFAM |  | **Activator** |
| Spo0A | 16079478 | OmpR | BG10765 | Medium | DBTBS | SUPFAM |  | **Dual** |
| SpoIIID | 16080695 | DeoR | BG10408 | Medium |  | SUPFAM | HMM-*ad hoc* | **Activator** |
| SpoVT | 16077124 | AbrB | BG10119 | Medium | DBTBS | SUPFAM |  | **Dual** |
| TenA | 16078230 | TenA | BG10791 | Medium | DBTBS | SUPFAM |  | **Activator** |
| TnrA | 16078396 | MerR | BG11805 | High | DBTBS | SUPFAM | HMM-*ad hoc* | **Dual** |
| TreR | 16077849 | GntR | BG11011 | High | DBTBS | SUPFAM | HMM-*ad hoc* | **Repressor** |
| Xpf | 16078321 | Xpf | BG10998 | Medium | DBTBS | SUPFAM |  | **Unknown** |
| Xre | 16078316 | Xre | BG10994 | Medium | DBTBS | SUPFAM |  | **Repressor** |
| XylR | 16078822 | ROK | BG11986 | Medium | DBTBS |  | HMM-*ad hoc* | **Repressor** |
| YazB | 16077148 | Xre | BG12701 | Medium | DBTBS | SUPFAM |  | ***Repressor*** |
| YbbB | 16077232 | AraC | BG10834 | High | DBTBS | SUPFAM | HMM-*ad hoc* | ***Dual*** |
| YbbH | 16077237 | RpiR | BG11569 | High | DBTBS | SUPFAM | HMM-*ad hoc* | ***Activator*** |
| YbdJ | 16077269 | OmpR | BG12723 | High | DBTBS | SUPFAM | HMM-*ad hoc* | **Activator** |
| YbfA | 16077285 | MarR | BG12732 | High | DBTBS | SUPFAM | HMM-*ad hoc* | **Repressor** |
| YbfI | 16077291 | AraC | BG12738 | High | DBTBS | SUPFAM | HMM-*ad hoc* | ***Dual*** |
| YbfP | 16077301 | AraC | BG12744 | High | DBTBS | SUPFAM | HMM-*ad hoc* | ***Dual*** |
| YbgA | 16077306 | GntR | BG12747 | High | DBTBS | SUPFAM | HMM-*ad hoc* | **Repressor** |
| YcbG | 16077319 | GntR | BG11162 | High | DBTBS | SUPFAM | HMM-*ad hoc* | **Repressor** |
| YcbL | 16077324 | LytTR | BG11167 | High | DBTBS | SUPFAM | HMM-*ad hoc* | **Unknown** |
| YccF | 16077341 | Psq | BG12756 | Medium | DBTBS | SUPFAM |  | **Unknown** |
| YccH | 16077343 | LytTR | BG11998 | Medium | DBTBS | SUPFAM |  | **Unknown** |
| YceK | 16077366 | ArsR | BG12775 | High | DBTBS | SUPFAM | HMM-*ad hoc* | **Repressor** |
| YcgE | 16077377 | MarR | BG12003 | Medium | DBTBS | SUPFAM |  | **Repressor** |
| YcgK | 16077386 | LysR | BG12009 | High | DBTBS | SUPFAM | HMM-*ad hoc* | **Dual** |
| YclA | 16077430 | LysR | BG12022 | High | DBTBS | SUPFAM | HMM-*ad hoc* | ***Dual*** |
| YcnC | 16077453 | **LysR`** | BG12039 | High | DBTBS | SUPFAM | HMM-*ad hoc* | **Repressor** |
| YcnK | 16077464 | DeoR | Bsu0397 | Medium |  | SUPFAM | HMM-*ad hoc* | ***Repressor*** |
| YcxD | 16077425 | GntR | BG10175 | Medium |  | SUPFAM | HMM-*ad hoc* | ***Repressor*** |
| YczG | 16077456 | ArsR | BG12781 | High | DBTBS | SUPFAM | HMM-*ad hoc* | **Repressor** |
| YdcH | 16077544 | MarR | BG12095 | High | DBTBS | SUPFAM | HMM-*ad hoc* | **Repressor** |
| YdcN | 16077549 | Xre | BG12101 | Medium | DBTBS | SUPFAM |  | ***Repressor*** |
| YdeC | 16077582 | AraC | BG12130 | High | DBTBS | SUPFAM | HMM-*ad hoc* | ***Dual*** |
| YdeE | 16077584 | AraC | BG12132 | High | DBTBS | SUPFAM | HMM-*ad hoc* | ***Dual*** |
| YdeF | 16077585 | GntR | BG12133 | High | DBTBS | SUPFAM | HMM-*ad hoc* | **Repressor** |
| YdeL | 16077591 | GntR | BG12139 | High | DBTBS | SUPFAM | HMM-*ad hoc* | **Repressor** |
| YdeP | 16077596 | DUF24 | BG12143 | High | DBTBS | SUPFAM | HMM-*ad hoc* | **Unknown** |
| YdeS | 16077599 | TetR | BG12146 | High | DBTBS | SUPFAM | HMM-*ad hoc* | **Repressor** |
| YdeT | 16077600 | ArsR | BG12147 | High | DBTBS | SUPFAM | HMM-*ad hoc* | **Repressor** |
| YdfD | 16077604 | GntR | BG12151 | High | DBTBS | SUPFAM | HMM-*ad hoc* | **Repressor** |
| YdfF | 16077606 | ArsR | BG12153 | Medium | DBTBS | SUPFAM |  | **Repressor** |
| YdfI | 16077609 | GerE | BG12156 | High | DBTBS | SUPFAM | HMM-*ad hoc* | **Activator** |
| YdfL | 16077613 | MerR | BG12159 | High | DBTBS | SUPFAM | HMM-*ad hoc* | **Repressor** |
| YdgC | 16077625 | TetR | BG12170 | High | DBTBS | SUPFAM | HMM-*ad hoc* | **Repressor** |
| YdgG | 16077631 | MarR | BG12174 | High | DBTBS | SUPFAM | HMM-*ad hoc* | **Repressor** |
| YdgJ | 16077634 | MarR | BG12177 | High | DBTBS | SUPFAM | HMM-*ad hoc* | **Repressor** |
| YdhC | 16077637 | GntR | BG12180 | High | DBTBS | SUPFAM | HMM-*ad hoc* | **Repressor** |
| YdhQ | 16077652 | GntR | BG12194 | High | DBTBS | SUPFAM | HMM-*ad hoc* | **Repressor** |
| YdhR | 16077653 | ROK | BG12195 | Medium | DBTBS | SUPFAM |  | **Repressor** |
| YdzF | 16077594 | MarR | BG12809 | Medium | DBTBS | SUPFAM |  | **Repressor** |
| YerO | 16077738 | TetR | BG12841 | High | DBTBS | SUPFAM | HMM-*ad hoc* | **Repressor** |
| YesN | 16077763 | AraC | BG12848 | Medium | DBTBS | SUPFAM |  | ***Dual*** |
| YesS | 16077768 | AraC | BG12853 | High | DBTBS | SUPFAM | HMM-*ad hoc* | ***Dual*** |
| YetL | 16077789 | MarR | BG12868 | High | DBTBS | SUPFAM | HMM-*ad hoc* | **Repressor** |
| YezC | 16077722 | AsnC | BG12874 | High | DBTBS | SUPFAM | HMM-*ad hoc* | ***Repressor*** |
| YezE | 16077886 | TetR | BG14194 | High | DBTBS | SUPFAM | HMM-*ad hoc* | **Repressor** |
| YfiF | 16077892 | AraC | BG11853 | High | DBTBS | SUPFAM | HMM-*ad hoc* | ***Dual*** |
| YfiK | 16077897 | GerE | BG12216 | High | DBTBS | SUPFAM | HMM-*ad hoc* | **Activator** |
| YfiR | 16077904 | TetR | BG12894 | High | DBTBS | SUPFAM | HMM-*ad hoc* | **Repressor** |
| YfiV | 16077908 | MarR | BG12898 | High | DBTBS | SUPFAM | HMM-*ad hoc* | **Repressor** |
| YfmP | 16077806 | MerR | BG12967 | High | DBTBS | SUPFAM | HMM-*ad hoc* | **Repressor** |
| YhbI | 16077964 | MarR | BG13003 | High | DBTBS | SUPFAM | HMM-*ad hoc* | **Repressor** |
| YhcB | 16077967 | WrbA | BG11580 | High | DBTBS | SUPFAM | HMM-*ad hoc* | **Repressor** |
| YhcF | 16077971 | GntR | BG11584 | High | DBTBS | SUPFAM | HMM-*ad hoc* | **Repressor** |
| YhcZ | 16077998 | GerE | BG13006 | High | DBTBS | SUPFAM | HMM-*ad hoc* | **Activator** |
| YhdE | 16078003 | Rrf2 | BG13011 | High | DBTBS | SUPFAM | HMM-*ad hoc* | **Unknown** |
| YhdI | 16078013 | GntR | BG13015 | High | DBTBS | SUPFAM | HMM-*ad hoc* | **Repressor** |
| YhdQ | 16078021 | MerR | BG13023 | High | DBTBS | SUPFAM | HMM-*ad hoc* | **Repressor** |
| YhgD | 16078079 | TetR | BG10432 | High | DBTBS | SUPFAM | HMM-*ad hoc* | **Repressor** |
| YhjH | 16078115 | MarR | BG13074 | Medium | DBTBS | SUPFAM |  | **Repressor** |
| YhjM | 16078120 | LacI | BG13079 | High | DBTBS | SUPFAM | HMM-*ad hoc* | **Repressor** |
| YisR | 16078146 | AraC | BG11064 | High | DBTBS | SUPFAM | HMM-*ad hoc* | ***Dual*** |
| YisV | 16078151 | GntR | BG13101 | Medium | DBTBS | SUPFAM |  | **Repressor** |
| YkoG | 16078390 | OmpR | BG13252 | Medium | DBTBS |  | HMM-*ad hoc* | **Activator** |
| YkoM | 16078399 | MarR | BG13258 | High | DBTBS | SUPFAM | HMM-*ad hoc* | **Repressor** |
| YkvE | 16078431 | MarR | BG13310 | High | DBTBS | SUPFAM | HMM-*ad hoc* | **Repressor** |
| YkvN | 16078440 | DUF24 | BG13316 | Medium | DBTBS | SUPFAM |  | **Unknown** |
| YkvZ | 16078451 | LacI | BG13327 | High | DBTBS | SUPFAM | HMM-*ad hoc* | **Repressor** |
| YmfC | 16078744 | GntR | BG13423 | High | DBTBS | SUPFAM | HMM-*ad hoc* | ***Repressor*** |
| YneI | 16078857 | OmpR | BG11250 | Medium | DBTBS | SUPFAM |  | **Activator** |
| YoaU | 16078936 | LysR | BG13491 | High | DBTBS | SUPFAM | HMM-*ad hoc* | **Dual** |
| YobD | 16078945 | Xre | BG13497 | High | DBTBS | SUPFAM | HMM-*ad hoc* | ***Repressor*** |
| YobQ | 16078965 | AraC | BG13508 | High | DBTBS | SUPFAM | HMM-*ad hoc* | ***Dual*** |
| YodB | 16079012 | ArsR | BG13531 | Medium | DBTBS | SUPFAM |  | ***Repressor*** |
| YofA | 16078903 | LysR | BG13552 | High | DBTBS | SUPFAM | HMM-*ad hoc* | ***Dual*** |
| YonN | 16079164 | IHF | BG13625 | Low |  |  | HMM-*ad hoc* | **Unknown** |
| YonR | 16079161 | Xre | BG13628 | Medium |  | SUPFAM | HMM-*ad hoc* | ***Repressor*** |
| YopO | 16079141 | Xre | BG13648 | Medium | DBTBS | SUPFAM |  | ***Repressor*** |
| YopS | 16079137 | Xre | BG13652 | Medium | DBTBS | SUPFAM |  | ***Repressor*** |
| YozA | 16078972 | ArsR | BG13748 | High | DBTBS | SUPFAM | HMM-*ad hoc* | **Repressor** |
| YozG | 16078934 | Xre | BG13754 | Medium | DBTBS | SUPFAM |  | ***Repressor*** |
| YpoP | 16079229 | MarR | BG11626 | Medium | DBTBS | SUPFAM |  | ***Repressor*** |
| YqaE | 16079688 | Xre | BG11256 | Medium | DBTBS | SUPFAM |  | ***Repressor*** |
| YqaF | 16079687 | Xre | BG11257 | Medium | DBTBS | SUPFAM |  | ***Repressor*** |
| YqaG | 16079685 | Xre | BG11258 | Medium | DBTBS | SUPFAM |  | ***Repressor*** |
| YraB | 16079754 | MerR | BG13777 | High | DBTBS | SUPFAM | HMM-*ad hoc* | **Repressor** |
| YraN | 16079740 | LysR | BG12278 | High | DBTBS | SUPFAM | HMM-*ad hoc* | **Dual** |
| YrdQ | 16079716 | LysR | BG12288 | High | DBTBS | SUPFAM | HMM-*ad hoc* | **Dual** |
| YrhI | 16079771 | TetR | BG12298 | High | DBTBS | SUPFAM | HMM-*ad hoc* | **Repressor** |
| YrkP | 16079696 | OmpR | BG11782 | High | DBTBS | SUPFAM | HMM-*ad hoc* | **Activator** |
| YrxA | 16079841 | YrxA | BG10865 | Medium | DBTBS |  | HMM-*ad hoc* | **Unknown** |
| YrzC | 16079806 | Rrf2 | BG13813 | High | DBTBS | SUPFAM | HMM-*ad hoc* | **Unknown** |
| YsiA | 16079907 | TetR | BG12330 | High | DBTBS | SUPFAM | HMM-*ad hoc* | ***Repressor*** |
| YsmB | 16079892 | MarR | BG12332 | High | DBTBS | SUPFAM | HMM-*ad hoc* | ***Repressor*** |
| YtcD | 16079955 | DUF24 | BG13831 | Medium | DBTBS | SUPFAM |  | **Unknown** |
| YtdP | 16080067 | AraC | BG13838 | High | DBTBS | SUPFAM | HMM-*ad hoc* | ***Dual*** |
| YtlI | 16079992 | LysR | BG13877 | Medium |  | SUPFAM | HMM-*ad hoc* | **Dual** |
| YtrA | 16080098 | GntR | BG13911 | Medium |  | SUPFAM | HMM-*ad hoc* | **Repressor** |
| YttP | 16080015 | TetR | BG13927 | Medium |  | SUPFAM | HMM-*ad hoc* | **Repressor** |
| YtzE | 16080054 | DeoR | BG13939 | Medium |  | SUPFAM | HMM-*ad hoc* | **Repressor** |
| YufM | 16080205 | OmpR | BG12348 | Medium | DBTBS | SUPFAM |  | **Activator** |
| YulB | 16080173 | DeoR | BG12384 | High | DBTBS | SUPFAM | HMM-*ad hoc* | **Repressor** |
| YurK | 16080309 | GntR | BG13997 | High | DBTBS | SUPFAM | HMM-*ad hoc* | **Repressor** |
| YurP | 16080314 | RpiR | BG14002 | Medium |  | SUPFAM | HMM-*ad hoc* | ***Activator*** |
| YusO | 16080339 | MarR | BG14027 | High | DBTBS | SUPFAM | HMM-*ad hoc* | **Repressor** |
| YusT | 16080344 | LysR | BG14032 | High | DBTBS | SUPFAM | HMM-*ad hoc* | **Dual** |
| YuxN | 16080356 | TetR | BG11072 | High | DBTBS | SUPFAM | HMM-*ad hoc* | **Repressor** |
| YvaF | 16080411 | TetR | BG14062 | High | DBTBS | SUPFAM | HMM-*ad hoc* | **Repressor** |
| YvaN | 16080419 | Xre | BG14069 | Medium | DBTBS | SUPFAM |  | ***Repressor*** |
| YvaO | 16080420 | Xre | BG14070 | Medium | DBTBS | SUPFAM |  | ***Repressor*** |
| YvaP | 16080421 | ArsR | BG14071 | Medium | DBTBS | SUPFAM |  | ***Repressor*** |
| YvbA | 16080432 | ArsR | BG14078 | High | DBTBS | SUPFAM | HMM-*ad hoc* | ***Repressor*** |
| YvbU | 16080452 | LysR | BG14087 | High | DBTBS | SUPFAM | HMM-*ad hoc* | ***Dual*** |
| YvdE | 16080516 | LacI | BG12414 | Medium | DBTBS | SUPFAM |  | ***Repressor*** |
| YvdT | 16080501 | TetR | BG12428 | High | DBTBS | SUPFAM | HMM-*ad hoc* | ***Repressor*** |
| YvfI | 16080471 | GntR | BG12434 | Medium | DBTBS | SUPFAM |  | ***Repressor*** |
| YvfU | 16080459 | GerE | BG12446 | High | DBTBS | SUPFAM | HMM-*ad hoc* | ***Activator*** |
| YvkB | 16080573 | TetR | BG14113 | High | DBTBS | SUPFAM | HMM-*ad hoc* | ***Repressor*** |
| YvmB | 16080561 | MarR | BG11018 | Medium | DBTBS | SUPFAM |  | ***Repressor*** |
| YvnA | 16080558 | MarR | BG14122 | Medium | DBTBS | SUPFAM |  | ***Repressor*** |
| YvoA | 16080556 | GntR | BG14124 | High | DBTBS | SUPFAM | HMM-*ad hoc* | ***Repressor*** |
| YvqC | 16080361 | GerE | BG14133 | High | DBTBS | SUPFAM | HMM-*ad hoc* | ***Activator*** |
| YvzC | 16080418 | Xre | BG14159 | Medium | DBTBS | SUPFAM |  | ***Repressor*** |
| YwaE | 16080896 | MarR | BG10556 | High | DBTBS | SUPFAM | HMM-*ad hoc* | ***Repressor*** |
| YwbI | 16080882 | LysR | BG10570 | High | DBTBS | SUPFAM | HMM-*ad hoc* | ***Dual*** |
| YwcC | 16080817 | TetR | BG10579 | High | DBTBS | SUPFAM | HMM-*ad hoc* | ***Repressor*** |
| YwgB | 16080810 | Rrf2 | BG12454 | Medium | DBTBS |  | HMM-*ad hoc* | **Unknown** |
| YwhA | 16080807 | MarR | BG12455 | High | DBTBS | SUPFAM | HMM-*ad hoc* | **Repressor** |
| YwoH | 16080697 | MarR | BG12495 | High | DBTBS | SUPFAM | HMM-*ad hoc* | **Repressor** |
| YwqM | 16080669 | LysR | BG12517 | High | DBTBS | SUPFAM | HMM-*ad hoc* | **Dual** |
| YwrC | 16080664 | AsnC | BG12522 | High | DBTBS | SUPFAM | HMM-*ad hoc* | ***Repressor*** |
| YxaD | 16081053 | MarR | BG11106 | High | DBTBS | SUPFAM | HMM-*ad hoc* | **Repressor** |
| YxaF | 16081051 | TetR | BG11108 | High | DBTBS | SUPFAM | HMM-*ad hoc* | **Repressor** |
| YxbF | 16081036 | TetR | BG11356 | High | DBTBS | SUPFAM | HMM-*ad hoc* | **Repressor** |
| YxdJ | 16081017 | OmpR | BG11126 | High | DBTBS | SUPFAM | HMM-*ad hoc* | ***Activator*** |
| YxjL | 16080942 | GerE | BG11895 | High | DBTBS | SUPFAM | HMM-*ad hoc* | ***Activator*** |
| YxjO | 16080939 | LysR | BG11898 | High | DBTBS | SUPFAM | HMM-*ad hoc* | **Dual** |
| YyaN | 16081132 | MerR | BG10038 | High | DBTBS | SUPFAM | HMM-*ad hoc* | **Repressor** |
| YybA | 16081123 | MarR | BG10030 | High | DBTBS | SUPFAM | HMM-*ad hoc* | **Repressor** |
| YybE | 16081119 | LysR | BG10026 | Medium |  | SUPFAM | HMM-*ad hoc* | **Dual** |
| YybR | 16081106 | HxlR | BG10013 | Medium | DBTBS | SUPFAM |  | ***Activator*** |
| YycF | 16081093 | OmpR | BG10001 | High | DBTBS | SUPFAM | HMM-*ad hoc* | ***Activator*** |
| YydK | 16081065 | GntR | BG11484 | High | DBTBS | SUPFAM | HMM-*ad hoc* | **Repressor** |
| Zur (YqfV) | 16079565 | FUR | BG11668 | Medium | DBTBS |  | HMM-*ad hoc* | **Repressor** |
