# Supplementary material for: Identification and analysis of DNA-binding transcription factors in Bacillus subtilis and other Firmicutes- a genomic approach
Source: BMC Genomics. 2006 Jun 13;7:147. doi: 10.1186/1471-2164-7-147 (PMC1524751; doi:10.1186/1471-2164-7-147)
Supplement: Additional File 2 — Table S2. Functional description of regulatory families. Columns are as follows: Family name, number of members, regulatory role, HTH position, DNA-binding structure and physiological role. [file 1471-2164-7-147-S2.doc]

| **Family** | **Regulatory role** | **Regulated functions** | **Domains** | **DBD motif** | **Position** |
| --- | --- | --- | --- | --- | --- |
| AraC/XylS | Dual | Carbon metabolism, stress response and pathogenesis | 2 (4), **7 (3)**, 3 (2) | Homeodomain-like | C-terminal |
| ArgR | Dual | Arginine biosynthesis | **1 (2)** | winged HTH | N-terminal |
| ArsR | Repressor | Metal resistance | 9 (1) | winged HTH | Central |
| AsnC | Repressor | Amino acid biosynthesis | **4 (2)**,2 (1) | winged HTH | N-terminal |
| BirA | Repressor | Regulation of the biotin operon |  | winged HTH | N-terminal |
| CodY | Repressor | Regulation of srfA and comK genes, dpp operon, gabP gene and hut operon ure operon | **1 (2)** | winged HTH |  |
| Cold shock | Activator | Low-temperature resistance | **3 (1)** | RNA binding domain (CSD) | Variable |
| ComK | Dual | Final autoregulatory control switch prior to competence development | **1 (1)** | Helical domain ofsec23/24 | **N-terminal** |
| Crp | Activator | Global responses, catabolite repression and anaerobiosis. | **1 (2)** | winged HTH | C-terminal |
| CtsR | Repressor | Regulator of class III stress genes (clpC, III stress genes (clpC, clpP). | **1 (1)** | nd |  |
| DeoR | Repressor | Sugar catabolism | **6 (2),** 2 (1) | winged HTH | N-terminal |
| DnaA | Repressor | initiation and regulation of chromosomal replication |  | TrpR-like | C-terminal |
| DtxR | Dual |  | **1 (2)** | winged HTH |  |
| DUF24 | Unknown | Hypothetical transcriptional regulator | **3 (1)** | winged HTH | **N-terminal** |
| EBP | Activator | Nitrogen assimilation ( arginine. ornithine, isoleucine and valine utilization as nitrogen sources) | **1 (3), 1 (4),1 (5)** | Fis (HTH) | C-terminal |
| FrvR | Activator |  |  | winged HTH |  |
| Fur | Repressor | Ferric uptake regulator family | **3 (1)** | winged HTH |  |
| GalR/LacI | Repressor | Carbon source utilization. catabolite repression | **11 (2)** | classical HTH | N-terminal |
| GntR | Repressor | Carbon (threalose, arabinose, gluconate) metabolism | **18 (2)**,3 (1) | winged HTH | N-terminal |
| HrcA | Repressor | Regulation of class I heat-shock genes (dnaK, groESL | **1 (3)** | winged HTH |  |
| HxlR | Activator |  | **2 (1)** | winged HTH |  |
| IcIR | Repressor | Carbon metabolism, efflux pumps | **1 (2)** | winged HTH | N-terminal |
| IHF | Unknown |  |  | IHF-like |  |
| LexA | Repressor | Regulation of the SOS regulon (DNA-damage inducible genes) | **1 (2)** | winged HTH | N-terminal |
| LuxR | Activator | Quorum sensing, biosynthesis and metabolism, etc. Some members belong to the two-component system | **10 (2)** | C_terminal (HTH) | C-terminal |
| LysR | Dual | Carbon and nitrogen metabolism | **19 (2)** | winged HTH | N-terminal |
| LytTR | Unknown | attenuator role for lytABC and lytR expression | **3 (2)** | Nd |  |
| MarR | Repressor | Multiple antibiotic resistance | **23 (1)**,1 (2) | winged HTH | Central |
| MerR | Dual | Resistance and detoxification | **6 (1)**,4 (2) | Putative DNA-bindingdomain (HTH) | N-terminal |
| OmpR | Activator | Heavy metal and virulence (response regulator of a two-component system) | **13 (2)** | winged HTH | C-terminal |
| PadR | Repressor | repression of padC | **1 (1)** | winged HTH | N-terminal |
| PaiA | Repressor |  |  |  |  |
| PRD | Activator | Substrate-dependent induction and catabolite repression of bglPH. |  | nd |  |
| PrpD | Unknown |  |  | nd |  |
| Psq | Unknown | unknown | **1 (2)** | HTH (?) | C-terminal |
| PucR | Dual | Regulation of puc genes (purine degradation |  | Homeodomain-like |  |
| PurR | Repressor | Regulation of the purine operons |  | nd |  |
| Rok | Repressor(¿?) | regulation of the xylose operon (xylAB) |  | HTH (?) |  |
| RpiR | Activator | rpiB gene repression. Also involved in *als* operon repression | **3 (2)** | nd |  |
| Rrf2 | Unknown | unknown | **3 (1)** | nd | **N-terminal** |
| RsfA | Dual | Improve the efficiency of sporulation. Represses spoIIR |  | Homeodomain-like |  |
| SenS | **Unknown** |  |  |  |  |
| SpoVT_AbrB | Dual | Regulation of transition state genes; positive regulation of comK | **3 (1)** | Transcription state regulator |  |
| TenA | Activator | Activator of extracellular enzyme genes (aprE, nprE, phoA, sacB) | **1 (1)** | nd |  |
| TetR | Repressor | Biosynthesis of antibiotics, efflux pumps, osmotic stress, etc. | **16 (1),**2 (2) | Homeodomain-like | C-terminal |
| WrbA | Repressor |  |  | Flavoproteins |  |
| Xpf | **Unknown** |  |  |  |  |
| Xre | Repressor | regulation of post-exponential-phase responses genes, regulation of competence development and sporulation genes | **17 (1)** | Classical | N-terminal |
| YjhU | **Repressor** |  |  |  |  |
| YjhU_YdeW | Repressor |  | **1 (2)** | winged HTH |  |
| YrxA | **Unknown** |  |  |  |  |
