# Supplementary material for: Identification and analysis of DNA-binding transcription factors in Bacillus subtilis and other Firmicutes- a genomic approach
Source: BMC Genomics. 2006 Jun 13;7:147. doi: 10.1186/1471-2164-7-147 (PMC1524751; doi:10.1186/1471-2164-7-147)
Supplement: Additional File 3 — Table S3. Identification of TFs by Superfamily searches. Nomenclature: Gene ID, Ids from Superfamily (DBD), and Domain Position. [file 1471-2164-7-147-S3.doc]

| **ID** | **IDE_DOMAIN** | **DOMAIN LOCATION** |
| --- | --- | --- |
| 16080015 | 0007288 | 5-68 |
| 16081065 | 0003426 | 1-73 |
| 16080807 | 0006957 | 4-137 |
| 16077105 | 0002348 | 3-55 |
| 16079892 | 0006957 | 5-139 |
| 16080173 | 0007802 | 1-59 |
| 16080173 | 0007359 | 73-235 |
| 16080354 | 0004400 | 7-176 |
| 16077722 | 0007060 | 1-50 |
| 16080420 | 0003722 | 1-64 |
| 16079963 | 0004400 | 7-182 |
| 16077975 | 0001190 | 1-65 |
| 16080452 | 0000692 | 1-106 |
| 16080452 | 0000338 | 87-290 |
| 16079944 | 0000066 | 1-134 |
| 16077324 | 0004400 | 2-173 |
| 16078512 | 0002348 | 1-51 |
| 16077457 | 0003426 | 16-86 |
| 16077457 | 0007713 | 88-468 |
| 16080942 | 0004400 | 10-182 |
| 16080516 | 0002240 | 1-58 |
| 16080516 | 0004784 | 71-315 |
| 16077337 | 0007288 | 5-74 |
| 16081028 | 0007802 | 5-54 |
| 16081028 | 0007359 | 71-234 |
| 16079458 | 0002523 | 3-84 |
| 16079458 | 0000934 | 121-277 |
| 16080664 | 0007060 | 12-71 |
| 16080664 | 0007062 | 73-151 |
| 16079481 | 0007208 | 3-78 |
| 16079481 | 0004975 | 80-149 |
| 16077886 | 0003833 | 58-252 |
| 16077591 | 0003426 | 12-85 |
| 16077591 | 0000672 | 70-462 |
| 16080078 | 0004873 | 2-59 |
| 16080078 | 0004784 | 65-338 |
| 16077634 | 0006957 | 24-157 |
| 16081139 | 0002240 | 1-58 |
| 16081139 | 0001073 | 58-295 |
| 16077124 | 0002348 | 1-54 |
| 16079907 | 0007288 | 10-75 |
| 16079270 | 0004873 | 10-65 |
| 16079270 | 0004784 | 67-338 |
| 16078003 | 0006466 | 3-65 |
| 16080411 | 0007288 | 3-68 |
| 16077232 | 0001607 | 216-264 |
| 16077232 | 0000887 | 165-214 |
| 16077232 | 0002244 | 263-524 |
| 16077249 | 0001607 | 151-199 |
| 16077249 | 0000887 | 97-150 |
| 16077249 | 0000246 | 17-109 |
| 16079688 | 0003722 | 2-67 |
| 16078151 | 0003426 | 13-81 |
| 16078151 | 0005091 | 95-230 |
| 16077343 | 0004400 | 6-164 |
| 16080655 | 0000692 | 1-98 |
| 16080655 | 0000338 | 87-285 |
| 16077892 | 0000887 | 187-240 |
| 16077892 | 0001607 | 242-287 |
| 16077892 | 0007789 | 11-97 |
| 16079806 | 0006466 | 4-67 |
| 16081087 | 0006106 | 139-413 |
| 16081087 | 0002500 | 12-120 |
| 16077599 | 0007288 | 13-64 |
| 16077625 | 0007288 | 7-74 |
| 16078965 | 0000888 | 186-236 |
| 16078965 | 0001606 | 140-183 |
| 16078965 | 0007789 | 23-78 |
| 16077606 | 0004673 | 1-89 |
| 16080817 | 0000692 | 1-105 |
| 16080817 | 0000338 | 89-291 |
| 16078808 | 0002523 | 9-78 |
| 16080419 | 0000251 | 1-65 |
| 16078399 | 0006957 | 15-143 |
| 16077306 | 0003426 | 1-73 |
| 16077789 | 0006957 | 28-160 |
| 16077291 | 0001607 | 220-267 |
| 16077291 | 0000887 | 170-216 |
| 16077291 | 0005083 | 9-158 |
| 16077319 | 0003426 | 15-83 |
| 16077366 | 0004673 | 5-98 |
| 16080558 | 0006957 | 44-151 |
| 16078680 | 0004673 | 189-256 |
| 16079893 | 0006025 | 8-70 |
| 16079252 | 0001190 | 1-66 |
| 16078744 | 0003426 | 7-79 |
| 16079685 | 0000251 | 6-63 |
| 16078063 | 0006957 | 27-131 |
| 16078013 | 0003426 | 12-85 |
| 16078013 | 0000672 | 102-462 |
| 16080421 | 0004673 | 83-170 |
| 16078146 | 0000888 | 232-280 |
| 16078146 | 0001606 | 184-228 |
| 16077768 | 0001607 | 708-755 |
| 16077768 | 0000887 | 659-707 |
| 16079720 | 0000692 | 1-104 |
| 16079720 | 0000338 | 85-294 |
| 16080669 | 0000692 | 1-104 |
| 16080669 | 0000338 | 86-293 |
| 16081057 | 0003426 | 16-81 |
| 16077584 | 0001607 | 57-104 |
| 16077584 | 0001606 | 7-56 |
| 16077584 | 0001608 | 124-290 |
| 16081053 | 0006957 | 5-139 |
| 16080067 | 0001607 | 719-770 |
| 16080067 | 0000887 | 670-716 |
| 16081093 | 0004400 | 7-178 |
| 16080784 | 0002975 | 146-223 |
| 16080784 | 0003429 | 12-145 |
| 16077631 | 0006957 | 7-141 |
| 16078440 | 0004673 | 1-82 |
| 16080361 | 0004400 | 6-175 |
| 16081132 | 0006895 | 3-125 |
| 16078120 | 0004144 | 1-55 |
| 16078120 | 0004784 | 59-329 |
| 16078380 | 0006957 | 8-141 |
| 16079711 | 0002523 | 6-112 |
| 16077464 | 0007826 | 1-55 |
| 16077806 | 0002523 | 1-130 |
| 16077477 | 0007826 | 5-78 |
| 16077477 | 0007674 | 79-246 |
| 16079771 | 0007288 | 10-76 |
| 16079141 | 0004526 | 1-62 |
| 16077904 | 0007288 | 12-77 |
| 16080602 | 0004400 | 8-191 |
| 16078934 | 0000553 | 3-69 |
| 16080026 | 0004873 | 4-59 |
| 16080026 | 0004784 | 61-332 |
| 16080309 | 0003426 | 7-80 |
| 16077596 | 0004673 | 13-95 |
| 16080098 | 0003426 | 9-77 |
| 16080054 | 0007826 | 8-71 |
| 16081123 | 0006957 | 1-133 |
| 16077998 | 0004400 | 5-174 |
| 16078008 | 0000692 | 1-105 |
| 16078008 | 0000338 | 86-257 |
| 16077237 | 0003833 | 60-259 |
| 16080882 | 0000692 | 1-105 |
| 16080882 | 0000338 | 90-298 |
| 16077269 | 0004451 | 118-217 |
| 16077269 | 0000066 | 1-133 |
| 16078396 | 0006895 | 15-103 |
| 16079955 | 0004673 | 1-85 |
| 16078302 | 0002240 | 1-58 |
| 16078302 | 0004784 | 59-328 |
| 16080683 | 0007802 | 1-60 |
| 16080683 | 0007359 | 75-234 |
| 16077897 | 0004400 | 6-183 |
| 16077908 | 0006957 | 44-150 |
| 16077579 | 0001190 | 1-65 |
| 16077613 | 0002523 | 2-120 |
| 16077613 | 0000934 | 162-270 |
| 16080356 | 0007288 | 3-72 |
| 16079012 | 0004673 | 1-83 |
| 16078431 | 0006957 | 1-135 |
| 16077148 | 0000251 | 1-68 |
| 16078115 | 0006957 | 10-155 |
| 16078147 | 0002240 | 1-59 |
| 16078147 | 0004784 | 60-336 |
| 16079725 | 0006245 | 5-68 |
| 16079725 | 0007062 | 66-147 |
| 16080450 | 0003426 | 23-96 |
| 16080450 | 0004784 | 101-378 |
| 16080501 | 0007288 | 10-76 |
| 16077386 | 0000692 | 1-105 |
| 16077386 | 0000338 | 86-323 |
| 16080561 | 0006957 | 27-151 |
| 16077585 | 0003426 | 24-97 |
| 16077585 | 0001176 | 79-464 |
| 16077604 | 0003426 | 11-79 |
| 16077604 | 0005529 | 104-459 |
| 16080713 | 0006895 | 3-105 |
| 16077600 | 0004673 | 1-81 |
| 16079229 | 0006957 | 1-137 |
| 16078980 | 0004400 | 6-162 |
| 16078972 | 0004673 | 13-103 |
| 16080896 | 0006957 | 40-166 |
| 16077964 | 0006957 | 11-141 |
| 16080939 | 0000692 | 1-105 |
| 16080939 | 0000338 | 86-289 |
| 16079992 | 0000692 | 1-104 |
| 16079992 | 0000338 | 85-298 |
| 16079740 | 0000692 | 1-104 |
| 16079740 | 0000338 | 86-284 |
| 16078907 | 0000692 | 1-105 |
| 16078907 | 0000338 | 87-295 |
| 16080697 | 0006957 | 1-135 |
| 16081017 | 0004400 | 6-168 |
| 16077544 | 0006957 | 7-128 |
| 16078903 | 0000692 | 1-101 |
| 16078903 | 0000338 | 85-283 |
| 16077901 | 0004673 | 5-89 |
| 16080573 | 0007288 | 3-72 |
| 16077572 | 0007060 | 2-61 |
| 16078770 | 0007288 | 9-75 |
| 16080432 | 0004673 | 1-84 |
| 16077285 | 0006957 | 12-138 |
| 16077285 | 0000663 | 162-302 |
| 16079716 | 0000692 | 1-100 |
| 16079716 | 0000338 | 88-282 |
| 16077492 | 0006245 | 1-66 |
| 16077492 | 0007062 | 63-121 |
| 16078316 | 0000251 | 1-64 |
| 16080994 | 0002975 | 15-53 |
| 16080644 | 0004144 | 1-54 |
| 16080644 | 0004784 | 59-322 |
| 16079478 | 0002710 | 149-266 |
| 16079478 | 0007720 | 1-124 |
| 16080556 | 0003426 | 8-81 |
| 16081036 | 0007288 | 8-72 |
| 16081036 | 0007288 | 202-266 |
| 16077377 | 0006957 | 8-141 |
| 16081051 | 0007288 | 6-76 |
| 16079466 | 0002460 | 577-686 |
| 16079466 | 0001264 | 1-96 |
| 16079466 | 0005819 | 17-40,391-599 |
| 16079466 | 0002500 | 102-219 |
| 16079466 | 0002500 | 223-340 |
| 16079517 | 0000552 | 74-108 |
| 16079517 | 0000553 | 1-67 |
| 16081119 | 0000692 | 3-91 |
| 16081119 | 0000338 | 73-278 |
| 16077738 | 0007288 | 3-72 |
| 16079161 | 0003722 | 2-76 |
| 16079696 | 0004400 | 7-172 |
| 16080491 | 0000553 | 1-63 |
| 16079754 | 0002523 | 1-116 |
| 16077456 | 0004673 | 3-94 |
| 16077849 | 0003426 | 2-75 |
| 16079137 | 0000553 | 10-69 |
| 16077594 | 0004673 | 1-91 |
| 16078945 | 0000251 | 1-69 |
| 16079508 | 0000567 | 1-62 |
| 16079508 | 0000840 | 63-136 |
| 16077877 | 0006106 | 292-542 |
| 16077877 | 0007674 | 67-203 |
| 16077582 | 0001607 | 240-288 |
| 16077582 | 0001606 | 186-239 |
| 16077582 | 0007481 | 22-115,166-193 |
| 16081106 | 0004673 | 6-91 |
| 16079634 | 0004673 | 16-89 |
| 16080344 | 0000692 | 1-101 |
| 16080344 | 0000338 | 87-281 |
| 16078451 | 0004873 | 3-57 |
| 16078451 | 0004784 | 59-321 |
| 16078390 | 0004400 | 8-176 |
| 16079687 | 0003722 | 4-74 |
| 16080459 | 0004400 | 6-165 |
| 16078502 | 0007826 | 1-73 |
| 16078502 | 0007359 | 73-231 |
| 16079369 | 0004400 | 11-179 |
| 16077430 | 0000692 | 1-103 |
| 16077430 | 0000338 | 87-290 |
| 16080470 | 0004144 | 1-57 |
| 16080470 | 0004784 | 70-328 |
| 16077513 | 0002975 | 177-209 |
| 16077513 | 0004400 | 10-187 |
| 16079416 | 0003722 | 4-64 |
| 16077425 | 0003426 | 1-73 |
| 16077425 | 0005091 | 74-438 |
| 16078936 | 0000692 | 2-108 |
| 16078936 | 0000338 | 89-286 |
| 16077573 | 0006245 | 3-66 |
| 16077573 | 0007062 | 63-123 |
| 16078848 | 0003691 | 1-72 |
| 16078848 | 0006529 | 80-202 |
| 16077609 | 0004400 | 6-174 |
| 16080339 | 0006957 | 5-138 |
| 16077637 | 0003426 | 9-81 |
| 16077652 | 0003426 | 1-73 |
| 16078079 | 0007288 | 4-73 |
| 16078478 | 0000692 | 1-105 |
| 16078478 | 0000338 | 87-290 |
| 16077971 | 0003426 | 8-76 |
| 16080418 | 0003894 | 1-76 |
| 16078021 | 0002523 | 10-118 |
| 16077763 | 0001607 | 310-361 |
| 16077763 | 0001606 | 254-309 |
| 16077763 | 0004400 | 6-192 |
| 16077301 | 0001607 | 57-104 |
| 16077301 | 0001606 | 7-55 |
| 16077453 | 0007288 | 3-64 |
| 16080219 | 0004400 | 6-179 |
| 16077416 | 0004673 | 1-88 |
| 16077549 | 0003722 | 1-66 |
