# Supplementary material for: Identification and analysis of DNA-binding transcription factors in Bacillus subtilis and other Firmicutes- a genomic approach
Source: BMC Genomics. 2006 Jun 13;7:147. doi: 10.1186/1471-2164-7-147 (PMC1524751; doi:10.1186/1471-2164-7-147)
Supplement: Additional File 4 — Table S4. Identification of TFs by HMM family specific. Columns are as follow: Gene ID, Family name, start and end position domains in the TF, bit and E-value scores. [file 1471-2164-7-147-S4.doc]

| **ID** | **Family** | **start** | **end** | **Bits** | **E-value** |
| --- | --- | --- | --- | --- | --- |
| 16077232 | AraC | 214 | 264 | 69.8 | 7.5e-16 |
| 16077249 | AraC | 98 | 150 | 51.5 | 1.1e-10 |
| 16077291 | AraC | 167 | 219 | 37.4 | 6.8e-07 |
| 16077301 | AraC | 56 | 106 | 37.1 | 8.1e-07 |
| 16077582 | AraC | 239 | 286 | 42.3 | 3.4e-08 |
| 16077584 | AraC | 4 | 56 | 32.3 | 1.6e-05 |
| 16077768 | AraC | 707 | 756 | 48.9 | 5.6e-10 |
| 16077892 | AraC | 240 | 289 | 56.7 | 4.5e-12 |
| 16078146 | AraC | 231 | 281 | 43.3 | 1.8e-08 |
| 16078965 | AraC | 185 | 235 | 37.5 | 6.4e-07 |
| 16080067 | AraC | 718 | 768 | 40.6 | 9.7e-08 |
| 16079481 | ArgR | 1 | 67 | 47.1 | 1.5e-11 |
| 16077366 | ArsR | 14 | 76 | 25.5 | 0.00062 |
| 16077456 | ArsR | 12 | 84 | 35.7 | 1.1e-06 |
| 16077600 | ArsR | 6 | 80 | 60.5 | 2.2e-13 |
| 16078972 | ArsR | 20 | 92 | 59.8 | 3.6e-13 |
| 16079634 | ArsR | 10 | 83 | 55.2 | 6.2e-12 |
| 16080432 | ArsR | 1 | 73 | 45.4 | 2.7e-09 |
| 16077492 | AsnC | 3 | 62 | 108.3 | 1.3e-31 |
| 16077572 | AsnC | 2 | 61 | 76.5 | 1.8e-21 |
| 16077573 | AsnC | 3 | 62 | 104.2 | 2.8e-30 |
| 16077722 | AsnC | 1 | 60 | 81.7 | 4e-23 |
| 16079725 | AsnC | 5 | 64 | 117.4 | 1.7e-34 |
| 16080664 | AsnC | 12 | 71 | 92.1 | 2e-26 |
| 16079301 | BirA | 1 | 72 | 142.6 | 9.1e-38 |
| 16077579 | Cold | 4 | 66 | 150.6 | 3.6e-40 |
| 16077975 | Cold | 4 | 66 | 142.5 | 1e-37 |
| 16079252 | Cold | 4 | 66 | 144.4 | 2.6e-38 |
| 16077464 | DeoR | 5 | 63 | 48.3 | 8.5e-11 |
| 16078502 | DeoR | 5 | 63 | 67.7 | 2.5e-16 |
| 16080054 | DeoR | 10 | 56 | 24.7 | 0.00045 |
| 16080173 | DeoR | 5 | 63 | 102.0 | 4.3e-26 |
| 16080683 | DeoR | 5 | 63 | 65.4 | 1.2e-15 |
| 16081028 | DeoR | 4 | 62 | 93.9 | 8.5e-24 |
| 16077069 | DnaA | 341 | 445 | 217.9 | 2e-60 |
| 16077877 | Fis | 559 | 599 | 59.5 | 9e-13 |
| 16079466 | Fis | 644 | 684 | 64.4 | 3.1e-14 |
| 16081087 | Fis | 415 | 455 | 65.3 | 1.7e-14 |
| 16080911 | FrvR | 1 | 63 | 25.0 | 0.00067 |
| 16077938 | Fur | 37 | 77 | 59.7 | 5.9e-14 |
| 16079409 | Fur | 37 | 77 | 65.5 | 1.3e-15 |
| 16079565 | Fur | 34 | 74 | 53.7 | 3e-12 |
| 16077306 | GntR | 3 | 73 | 87.0 | 4.5e-21 |
| 16077319 | GntR | 17 | 84 | 89.8 | 7e-22 |
| 16077425 | GntR | 2 | 72 | 94.5 | 2.8e-23 |
| 16077457 | GntR | 15 | 85 | 73.5 | 2.5e-17 |
| 16077585 | GntR | 26 | 96 | 57.9 | 5.1e-13 |
| 16077591 | GntR | 14 | 77 | 55.2 | 2.9e-12 |
| 16077604 | GntR | 13 | 83 | 71.7 | 7.8e-17 |
| 16077637 | GntR | 11 | 80 | 96.2 | 8.7e-24 |
| 16077652 | GntR | 2 | 72 | 62.1 | 3.6e-14 |
| 16077849 | GntR | 4 | 73 | 99.7 | 7.6e-25 |
| 16077971 | GntR | 10 | 80 | 61.3 | 5.9e-14 |
| 16078013 | GntR | 14 | 84 | 54.9 | 3.5e-12 |
| 16078744 | GntR | 9 | 79 | 118.7 | 1.5e-30 |
| 16080098 | GntR | 11 | 81 | 69.7 | 2.8e-16 |
| 16080309 | GntR | 9 | 79 | 120.7 | 3.5e-31 |
| 16080450 | GntR | 25 | 95 | 118.7 | 1.4e-30 |
| 16080556 | GntR | 10 | 80 | 109.6 | 7.7e-28 |
| 16081057 | GntR | 18 | 86 | 72.4 | 5.1e-17 |
| 16081065 | GntR | 2 | 72 | 92.8 | 9e-23 |
| 16077477 | IclR | 6 | 77 | 109.6 | 5.4e-28 |
| 16079164 | Ihf | 1 | 92 | 168.3 | 7.6e-49 |
| 16079336 | Ihf | 1 | 92 | 194.1 | 9.7e-57 |
| 16078120 | LacI | 1 | 56 | 96.2 | 9.5e-28 |
| 16078147 | LacI | 2 | 56 | 107.5 | 2.3e-31 |
| 16078302 | LacI | 1 | 55 | 98.4 | 1.8e-28 |
| 16078451 | LacI | 1 | 55 | 114.0 | 2e-33 |
| 16079270 | LacI | 8 | 64 | 98.4 | 1.8e-28 |
| 16080026 | LacI | 3 | 57 | 112.2 | 7.6e-33 |
| 16080078 | LacI | 1 | 48 | 67.0 | 2e-18 |
| 16080470 | LacI | 1 | 48 | 76.6 | 1.7e-21 |
| 16080516 | LacI | 1 | 55 | 109.0 | 8e-32 |
| 16080644 | LacI | 1 | 55 | 114.0 | 2e-33 |
| 16081139 | LacI | 1 | 55 | 112.4 | 6.6e-33 |
| 16078848 | LexA | 1 | 57 | 111.4 | 5.6e-32 |
| 16077609 | LuxR | 145 | 209 | 58.1 | 1.9e-13 |
| 16077897 | LuxR | 154 | 218 | 76.9 | 9.3e-19 |
| 16077998 | LuxR | 148 | 209 | 93.4 | 2.1e-23 |
| 16078980 | LuxR | 134 | 198 | 68.4 | 2.4e-16 |
| 16079893 | LuxR | 8 | 72 | 78.7 | 2.9e-19 |
| 16080219 | LuxR | 150 | 214 | 74.2 | 5.3e-18 |
| 16080361 | LuxR | 146 | 210 | 103.3 | 3.1e-26 |
| 16080459 | LuxR | 136 | 200 | 65.0 | 2.2e-15 |
| 16080602 | LuxR | 162 | 226 | 109.3 | 6.4e-28 |
| 16080942 | LuxR | 153 | 217 | 97.4 | 1.5e-24 |
| 16077386 | LysR | 1 | 77 | 106.9 | 1.5e-27 |
| 16077430 | LysR | 1 | 78 | 114.7 | 8.8e-30 |
| 16078008 | LysR | 1 | 77 | 102.0 | 3.5e-26 |
| 16078478 | LysR | 1 | 77 | 93.0 | 1.3e-23 |
| 16078903 | LysR | 1 | 77 | 127.8 | 1.6e-33 |
| 16078907 | LysR | 1 | 77 | 121.1 | 1.3e-31 |
| 16078936 | LysR | 3 | 80 | 101.4 | 5.4e-26 |
| 16079716 | LysR | 1 | 77 | 109.1 | 3.3e-28 |
| 16079720 | LysR | 1 | 77 | 108.6 | 4.7e-28 |
| 16079740 | LysR | 1 | 77 | 99.5 | 1.9e-25 |
| 16079992 | LysR | 1 | 76 | 105.8 | 3e-27 |
| 16080344 | LysR | 1 | 77 | 109.8 | 2.2e-28 |
| 16080452 | LysR | 1 | 78 | 116.4 | 2.8e-30 |
| 16080655 | LysR | 1 | 77 | 138.4 | 1.5e-36 |
| 16080669 | LysR | 1 | 77 | 99.2 | 2.2e-25 |
| 16080817 | LysR | 1 | 77 | 103.4 | 1.5e-26 |
| 16080882 | LysR | 1 | 77 | 124.5 | 1.4e-32 |
| 16080939 | LysR | 1 | 77 | 129.7 | 4.5e-34 |
| 16081119 | LysR | 1 | 63 | 92.0 | 2.6e-23 |
| 16077285 | MarR | 33 | 143 | 26.5 | 6.6e-05 |
| 16077544 | MarR | 12 | 108 | 25.4 | 0.00014 |
| 16077631 | MarR | 73 | 146 | 25.3 | 0.00015 |
| 16077634 | MarR | 60 | 160 | 66.9 | 2.2e-16 |
| 16077789 | MarR | 28 | 163 | 52.2 | 3.4e-12 |
| 16077908 | MarR | 67 | 141 | 43.2 | 1.2e-09 |
| 16077964 | MarR | 23 | 110 | 40.8 | 5.6e-09 |
| 16078380 | MarR | 33 | 105 | 34.0 | 5e-07 |
| 16078399 | MarR | 37 | 143 | 37.2 | 6.1e-08 |
| 16078431 | MarR | 7 | 140 | 29.2 | 1.2e-05 |
| 16079229 | MarR | 10 | 140 | 35.5 | 1.8e-07 |
| 16079892 | MarR | 10 | 144 | 42.6 | 1.8e-09 |
| 16080339 | MarR | 9 | 143 | 37.6 | 4.6e-08 |
| 16080697 | MarR | 7 | 132 | 62.0 | 5.6e-15 |
| 16080807 | MarR | 70 | 137 | 33.3 | 8e-07 |
| 16080896 | MarR | 82 | 169 | 36.9 | 7.3e-08 |
| 16081053 | MarR | 55 | 142 | 22.5 | 0.00093 |
| 16081123 | MarR | 2 | 136 | 155.6 | 1.1e-41 |
| 16077613 | MerR | 5 | 65 | 37.2 | 2.7e-06 |
| 16077806 | MerR | 4 | 64 | 75.9 | 1.1e-17 |
| 16078021 | MerR | 12 | 60 | 51.9 | 1.9e-10 |
| 16078396 | MerR | 14 | 59 | 47.9 | 2.9e-09 |
| 16078808 | MerR | 12 | 70 | 48.3 | 2.3e-09 |
| 16079711 | MerR | 9 | 68 | 28.6 | 0.00042 |
| 16079754 | MerR | 1 | 60 | 84.2 | 3.5e-20 |
| 16080713 | MerR | 3 | 62 | 40.8 | 3.1e-07 |
| 16081132 | MerR | 4 | 63 | 76.0 | 1e-17 |
| 16077269 | OmpR | 144 | 217 | 75.2 | 1.7e-17 |
| 16077324 | OmpR | 147 | 223 | 93.1 | 7.2e-23 |
| 16078390 | OmpR | 151 | 226 | 95.1 | 1.8e-23 |
| 16079369 | OmpR | 158 | 235 | 94.6 | 2.6e-23 |
| 16079696 | OmpR | 151 | 228 | 71.5 | 2.3e-16 |
| 16079963 | OmpR | 157 | 233 | 95.8 | 1.1e-23 |
| 16080354 | OmpR | 150 | 222 | 73.4 | 6.2e-17 |
| 16081017 | OmpR | 150 | 225 | 84.6 | 2.6e-20 |
| 16081093 | OmpR | 153 | 229 | 93.4 | 5.6e-23 |
| 16079469 | PrpD | 1 | 472 | 1224.5 | 0 |
| 16078822 | Rok | 1 | 20 | 26.3 | 0.00014 |
| 16077237 | RpiR | 3 | 276 | 500.3 | 1.9e-145 |
| 16077886 | RpiR | 1 | 68 | 53.5 | 1.9e-12 |
| 16080314 | RpiR | 76 | 121 | 30.4 | 5.5e-06 |
| 16077337 | TetR | 5 | 75 | 49.0 | 2.1e-11 |
| 16077453 | TetR | 3 | 74 | 45.2 | 2.5e-10 |
| 16077599 | TetR | 12 | 50 | 31.3 | 2.8e-06 |
| 16077625 | TetR | 7 | 73 | 45.1 | 2.8e-10 |
| 16077738 | TetR | 3 | 73 | 69.2 | 2.6e-17 |
| 16077904 | TetR | 12 | 65 | 38.0 | 3.1e-08 |
| 16078079 | TetR | 4 | 75 | 59.5 | 1.7e-14 |
| 16078770 | TetR | 9 | 77 | 91.1 | 1.1e-23 |
| 16079771 | TetR | 12 | 77 | 38.1 | 2.9e-08 |
| 16079907 | TetR | 4 | 77 | 61.1 | 5.8e-15 |
| 16080015 | TetR | 4 | 74 | 54.5 | 4.9e-13 |
| 16080356 | TetR | 3 | 61 | 67.5 | 8.2e-17 |
| 16080411 | TetR | 3 | 69 | 42.9 | 1.2e-09 |
| 16080501 | TetR | 10 | 77 | 60.8 | 7.5e-15 |
| 16080573 | TetR | 3 | 73 | 50.8 | 5.8e-12 |
| 16081051 | TetR | 6 | 54 | 34.8 | 2.6e-07 |
| 16077967 | WrbA | 1 | 174 | 283.5 | 5.3e-86 |
| 16078003 | YjeB | 1 | 145 | 304.7 | 7.1e-98 |
| 16079806 | YjeB | 2 | 93 | 91.1 | 1.7e-27 |
| 16080994 | YjhU_YdeW | 55 | 313 | 562.8 | 2.9e-164 |
