# Supplementary material for: Identification and analysis of DNA-binding transcription factors in Bacillus subtilis and other Firmicutes- a genomic approach
Source: BMC Genomics. 2006 Jun 13;7:147. doi: 10.1186/1471-2164-7-147 (PMC1524751; doi:10.1186/1471-2164-7-147)
Supplement: Additional File 5 — Table S5. Genomes analyzed in this work. Columns are as follows: Cellular division, order, Organism name, Taxonomic Identifier, Genome size (Mbp), number of TFs and Genome code used in the manuscript. [file 1471-2164-7-147-S5.doc]

| **Division** | **Order** | **Organism Name** | **Taxon ID** | **Genome size** | **Transcription**  **Factors** | **Code** |
| --- | --- | --- | --- | --- | --- | --- |
| Firmicutes | Bacillales | [Bacillus anthracis Ames](http://cmr.tigr.org/tigr-scripts/CMR/GenomePage.cgi?org=gba) | [198094](http://www.ncbi.nlm.nih.gov/Taxonomy/Browser/wwwtax.cgi?id=198094) | 5.22 Mb | 272 | Ban_A2012 |
| Firmicutes | Bacillales | [Bacillus anthracis Ames Ancestor](http://cmr.tigr.org/tigr-scripts/CMR/GenomePage.cgi?org=gb6) | [261594](http://www.ncbi.nlm.nih.gov/Taxonomy/Browser/wwwtax.cgi?id=261594) | 5.50 Mb | 273 | Ban |
| Firmicutes | Bacillales | [Bacillus anthracis Sterne](http://cmr.tigr.org/tigr-scripts/CMR/GenomePage.cgi?org=ntba05) | [260799](http://www.ncbi.nlm.nih.gov/Taxonomy/Browser/wwwtax.cgi?id=260799) | 5.22 Mb | 278 | Ban_0581 |
| Firmicutes | Bacillales | [Bacillus anthracis strain A2012](http://cmr.tigr.org/tigr-scripts/CMR/GenomePage.cgi?org=gbx) | [191218](http://www.ncbi.nlm.nih.gov/Taxonomy/Browser/wwwtax.cgi?id=191218) | 0.27 Mb | 250 | Ban_Sterne |
| Firmicutes | Bacillales | [Bacillus cereus 10987](http://cmr.tigr.org/tigr-scripts/CMR/GenomePage.cgi?org=gbc) | [222523](http://www.ncbi.nlm.nih.gov/Taxonomy/Browser/wwwtax.cgi?id=222523) | 5.43 Mb | 275 | Bce |
| Firmicutes | Bacillales | [Bacillus cereus ATCC14579](http://cmr.tigr.org/tigr-scripts/CMR/GenomePage.cgi?org=ntbc01) | [226900](http://www.ncbi.nlm.nih.gov/Taxonomy/Browser/wwwtax.cgi?id=226900) | 5.42 Mb | 260 | Bce_ATCC14579 |
| Firmicutes | Bacillales | [Bacillus cereus Zk](http://cmr.tigr.org/tigr-scripts/CMR/GenomePage.cgi?org=ntbc02) | [288681](http://www.ncbi.nlm.nih.gov/Taxonomy/Browser/wwwtax.cgi?id=288681) | 5.30 Mb | 292 | Bce_ZK |
| Firmicutes | Bacillales | [Bacillus clausii KSM-K16](http://cmr.tigr.org/tigr-scripts/CMR/GenomePage.cgi?org=ntbc03) | [66692](http://www.ncbi.nlm.nih.gov/Taxonomy/Browser/wwwtax.cgi?id=66692) | 4.30 Mb | 278 | Bcl |
| Firmicutes | Bacillales | [Bacillus halodurans C-125](http://cmr.tigr.org/tigr-scripts/CMR/GenomePage.cgi?org=ntbh01) | [272558](http://www.ncbi.nlm.nih.gov/Taxonomy/Browser/wwwtax.cgi?id=272558) | 4.20 Mb | 231 | Bha |
| Firmicutes | Bacillales | [Bacillus licheniformis ATCC 14580](http://cmr.tigr.org/tigr-scripts/CMR/GenomePage.cgi?org=ntbl03) | [279010](http://www.ncbi.nlm.nih.gov/Taxonomy/Browser/wwwtax.cgi?id=279010) | 4.22 Mb | 260 | Bli_14580 |
| Firmicutes | Bacillales | [Bacillus licheniformis ATCC14580 (DSM 13)](http://cmr.tigr.org/tigr-scripts/CMR/GenomePage.cgi?org=ntbl04) | [279010](http://www.ncbi.nlm.nih.gov/Taxonomy/Browser/wwwtax.cgi?id=279010) | 4.22 Mb | 266 | Bli |
| Firmicutes | Bacillales | [Bacillus subtilis 168](http://cmr.tigr.org/tigr-scripts/CMR/GenomePage.cgi?org=ntbs01) | [224308](http://www.ncbi.nlm.nih.gov/Taxonomy/Browser/wwwtax.cgi?id=224308) | 4.21 Mb | 237 | Bsu |
| Firmicutes | Bacillales | [Bacillus thuringiensis konkukian](http://cmr.tigr.org/tigr-scripts/CMR/GenomePage.cgi?org=ntbt02) | [281309](http://www.ncbi.nlm.nih.gov/Taxonomy/Browser/wwwtax.cgi?id=281309) | 5.22 Mb | 265 | Bth |
| Firmicutes | Bacillales | [Geobacillus kaustophilus HTA426](http://cmr.tigr.org/tigr-scripts/CMR/GenomePage.cgi?org=ntgk01) | [235909](http://www.ncbi.nlm.nih.gov/Taxonomy/Browser/wwwtax.cgi?id=235909) | 3.59 Mb | 134 | Gka |
| Firmicutes | Bacillales | [Listeria innocua CLIP 11262](http://cmr.tigr.org/tigr-scripts/CMR/GenomePage.cgi?org=ntli01) | [272626](http://www.ncbi.nlm.nih.gov/Taxonomy/Browser/wwwtax.cgi?id=272626) | 3.09 Mb | 160 | Lin |
| Firmicutes | Bacillales | [Listeria monocytogenes 4b F2365](http://cmr.tigr.org/tigr-scripts/CMR/GenomePage.cgi?org=blm) | [265669](http://www.ncbi.nlm.nih.gov/Taxonomy/Browser/wwwtax.cgi?id=265669) | 2.90 Mb | 162 | Lmo |
| Firmicutes | Bacillales | [Listeria monocytogenes EGD-e](http://cmr.tigr.org/tigr-scripts/CMR/GenomePage.cgi?org=ntlm01) | [169963](http://www.ncbi.nlm.nih.gov/Taxonomy/Browser/wwwtax.cgi?id=169963) | 2.94 Mb | 150 | Lmo_F2365 |
| Firmicutes | Bacillales | [Oceanobacillus iheyensis HTE831](http://cmr.tigr.org/tigr-scripts/CMR/GenomePage.cgi?org=ntoi01) | [221109](http://www.ncbi.nlm.nih.gov/Taxonomy/Browser/wwwtax.cgi?id=221109) | 3.63 Mb | 166 | Oih |
| Firmicutes | Bacillales | [Phytoplasma asteris Onion Yellows strain](http://cmr.tigr.org/tigr-scripts/CMR/GenomePage.cgi?org=ntoy01) | [100379](http://www.ncbi.nlm.nih.gov/Taxonomy/Browser/wwwtax.cgi?id=100379) | 0.86 Mb | 3 | Oye |
| Firmicutes | Bacillales | [Staphylococcus aureus subsp. aureus COL](http://cmr.tigr.org/tigr-scripts/CMR/GenomePage.cgi?org=gsa) | [93062](http://www.ncbi.nlm.nih.gov/Taxonomy/Browser/wwwtax.cgi?id=93062) | 2.81 Mb | 89 | Sau |
| Firmicutes | Bacillales | [Staphylococcus aureus subsp. aureus MRSA252](http://cmr.tigr.org/tigr-scripts/CMR/GenomePage.cgi?org=ntsa08) | [282458](http://www.ncbi.nlm.nih.gov/Taxonomy/Browser/wwwtax.cgi?id=282458) | 2.90 Mb | 91 | Sau_MSSA476 |
| Firmicutes | Bacillales | [Staphylococcus aureus Michigan VRSA](http://cmr.tigr.org/tigr-scripts/CMR/GenomePage.cgi?org=gvrsa) | [1280](http://www.ncbi.nlm.nih.gov/Taxonomy/Browser/wwwtax.cgi?id=1280) | 0.5 Mb | 94 | Sau_COL |
| Firmicutes | Bacillales | [Staphylococcus aureus Mu50](http://cmr.tigr.org/tigr-scripts/CMR/GenomePage.cgi?org=ntsa02) | [158878](http://www.ncbi.nlm.nih.gov/Taxonomy/Browser/wwwtax.cgi?id=158878) | 2.90 Mb | 95 | Sau_Mu50 |
| Firmicutes | Bacillales | [Staphylococcus aureus MW2](http://cmr.tigr.org/tigr-scripts/CMR/GenomePage.cgi?org=ntsa03) | [196620](http://www.ncbi.nlm.nih.gov/Taxonomy/Browser/wwwtax.cgi?id=196620) | 2.82 Mb | 93 | Sau_MW2 |
| Firmicutes | Bacillales | [Staphylococcus aureus N315](http://cmr.tigr.org/tigr-scripts/CMR/GenomePage.cgi?org=ntsa01) | [158879](http://www.ncbi.nlm.nih.gov/Taxonomy/Browser/wwwtax.cgi?id=158879) | 2.83 Mb | 95 | Sau_N315 |
| Firmicutes | Bacillales | [Staphylococcus epidermidis ATCC 12228](http://cmr.tigr.org/tigr-scripts/CMR/GenomePage.cgi?org=ntse02) | [176280](http://www.ncbi.nlm.nih.gov/Taxonomy/Browser/wwwtax.cgi?id=176280) | 2.49 Mb | 67 | Sep |
| Firmicutes | Bacillales | [Staphylococcus epidermidis RP62A](http://cmr.tigr.org/tigr-scripts/CMR/GenomePage.cgi?org=gse) | [176279](http://www.ncbi.nlm.nih.gov/Taxonomy/Browser/wwwtax.cgi?id=176279) | 2.64 Mb | 75 | Sep_RP62A |
| Firmicutes | Clostridia | [Clostridium acetobutylicum ATCC824](http://cmr.tigr.org/tigr-scripts/CMR/GenomePage.cgi?org=ntca01) | [272562](http://www.ncbi.nlm.nih.gov/Taxonomy/Browser/wwwtax.cgi?id=272562) | 4.13 Mb | 197 | Cac |
| Firmicutes | Clostridia | [Clostridium perfringens 13](http://cmr.tigr.org/tigr-scripts/CMR/GenomePage.cgi?org=ntcp03) | [195102](http://www.ncbi.nlm.nih.gov/Taxonomy/Browser/wwwtax.cgi?id=195102) | 3.08 Mb | 96 | Cpe |
| Firmicutes | Clostridia | [Clostridium tetani E88](http://cmr.tigr.org/tigr-scripts/CMR/GenomePage.cgi?org=ntct02) | [212717](http://www.ncbi.nlm.nih.gov/Taxonomy/Browser/wwwtax.cgi?id=212717) | 2.79 Mb | 104 | Cte |
| Firmicutes | Clostridia | [Thermoanaerobacter tengcongensis MB4(T)](http://cmr.tigr.org/tigr-scripts/CMR/GenomePage.cgi?org=nttt01) | [273068](http://www.ncbi.nlm.nih.gov/Taxonomy/Browser/wwwtax.cgi?id=273068) | 2.68 Mb | 83 | Tte |
| Firmicutes | Lactobacillales | [Enterococcus faecalis V583](http://cmr.tigr.org/tigr-scripts/CMR/GenomePage.cgi?org=gef) | [226185](http://www.ncbi.nlm.nih.gov/Taxonomy/Browser/wwwtax.cgi?id=226185) | 3.35 Mb | 144 | Efa |
| Firmicutes | Lactobacillales | [Lactobacillus acidophilus NCFM](http://cmr.tigr.org/tigr-scripts/CMR/GenomePage.cgi?org=ntla01) | [272621](http://www.ncbi.nlm.nih.gov/Taxonomy/Browser/wwwtax.cgi?id=272621) | 1.99 Mb | 53 | Lac |
| Firmicutes | Lactobacillales | [Lactobacillus johnsonii NCC 533](http://cmr.tigr.org/tigr-scripts/CMR/GenomePage.cgi?org=ntlj01) | [257314](http://www.ncbi.nlm.nih.gov/Taxonomy/Browser/wwwtax.cgi?id=257314) | 1.99 Mb | 55 | Ljo |
| Firmicutes | Lactobacillales | [Lactococcus lactis subsp. lactis IL1403](http://cmr.tigr.org/tigr-scripts/CMR/GenomePage.cgi?org=ntll01) | [272623](http://www.ncbi.nlm.nih.gov/Taxonomy/Browser/wwwtax.cgi?id=272623) | 2.36 Mb | 85 | Lla |
| Firmicutes | Lactobacillales | [Lactobacillus plantarum WCFS1](http://cmr.tigr.org/tigr-scripts/CMR/GenomePage.cgi?org=ntlp01) | [220668](http://www.ncbi.nlm.nih.gov/Taxonomy/Browser/wwwtax.cgi?id=220668) | 3.30 Mb | 162 | Lpl |
| Firmicutes | Lactobacillales | [Streptococcus agalactiae 2603V/R](http://cmr.tigr.org/tigr-scripts/CMR/GenomePage.cgi?org=gbs) | [208435](http://www.ncbi.nlm.nih.gov/Taxonomy/Browser/wwwtax.cgi?id=208435) | 2.16 Mb | 74 | Sag |
| Firmicutes | Lactobacillales | [Streptococcus agalactiae NEM316](http://cmr.tigr.org/tigr-scripts/CMR/GenomePage.cgi?org=ntsa04) | [211110](http://www.ncbi.nlm.nih.gov/Taxonomy/Browser/wwwtax.cgi?id=211110) | 2.21 Mb | 70 | Sag_NEM316 |
| Firmicutes | Lactobacillales | [Streptococcus mutans UA159](http://cmr.tigr.org/tigr-scripts/CMR/GenomePage.cgi?org=ntsm02) | [210007](http://www.ncbi.nlm.nih.gov/Taxonomy/Browser/wwwtax.cgi?id=210007) | 2.03 Mb | 84 | Smu |
| Firmicutes | Lactobacillales | [Streptococcus pneumoniae R6](http://cmr.tigr.org/tigr-scripts/CMR/GenomePage.cgi?org=ntsp02) | [171101](http://www.ncbi.nlm.nih.gov/Taxonomy/Browser/wwwtax.cgi?id=171101) | 2.03 Mb | 54 | Spn_R6 |
| Firmicutes | Lactobacillales | [Streptococcus pneumoniae TIGR4](http://cmr.tigr.org/tigr-scripts/CMR/GenomePage.cgi?org=bsp) | [170187](http://www.ncbi.nlm.nih.gov/Taxonomy/Browser/wwwtax.cgi?id=170187) | 2.16 Mb | 58 | Spn |
| Firmicutes | Lactobacillales | [Streptococcus pyogenes MGAS5005](http://cmr.tigr.org/tigr-scripts/CMR/GenomePage.cgi?org=ntsp10) | [293653](http://www.ncbi.nlm.nih.gov/Taxonomy/Browser/wwwtax.cgi?id=293653) | 1.83 Mb | 55 | Spy_GAS |
| Firmicutes | Lactobacillales | [Streptococcus pyogenes MGAS10394](http://cmr.tigr.org/tigr-scripts/CMR/GenomePage.cgi?org=ntsp11) | [286636](http://www.ncbi.nlm.nih.gov/Taxonomy/Browser/wwwtax.cgi?id=286636) | 1.89 Mb | 58 | Spy_MGAS10394 |
| Firmicutes | Lactobacillales | [Streptococcus pyogenes MGAS315](http://cmr.tigr.org/tigr-scripts/CMR/GenomePage.cgi?org=ntsp04) | [198466](http://www.ncbi.nlm.nih.gov/Taxonomy/Browser/wwwtax.cgi?id=198466) | 1.90 Mb | 63 | Spy |
| Firmicutes | Lactobacillales | [Streptococcus pyogenes MGAS8232](http://cmr.tigr.org/tigr-scripts/CMR/GenomePage.cgi?org=ntsp03) | [186103](http://www.ncbi.nlm.nih.gov/Taxonomy/Browser/wwwtax.cgi?id=186103) | 1.89 Mb | 61 | Spy_MGAS8232 |
| Firmicutes | Lactobacillales | [Streptococcus pyogenes SSI-1](http://cmr.tigr.org/tigr-scripts/CMR/GenomePage.cgi?org=ntsp06) | [193567](http://www.ncbi.nlm.nih.gov/Taxonomy/Browser/wwwtax.cgi?id=193567) | 1.89 Mb | 65 | Spy_SSI-1 |
| Firmicutes | Lactobacillales | [Streptococcus thermophilus CNRZ1066](http://cmr.tigr.org/tigr-scripts/CMR/GenomePage.cgi?org=ntst08) | [299768](http://www.ncbi.nlm.nih.gov/Taxonomy/Browser/wwwtax.cgi?id=299768) | 1.79 Mb | 37 | Sth |
| Firmicutes | Lactobacillales | [Streptococcus thermophilus LMG 18311](http://cmr.tigr.org/tigr-scripts/CMR/GenomePage.cgi?org=ntst09) | [264199](http://www.ncbi.nlm.nih.gov/Taxonomy/Browser/wwwtax.cgi?id=264199) | 1.79 Mb | 34 | Sth_18311 |
| Firmicutes | Mollicutes | [Mesoplasma florum L1](http://cmr.tigr.org/tigr-scripts/CMR/GenomePage.cgi?org=ntmf02) | [265311](http://www.ncbi.nlm.nih.gov/Taxonomy/Browser/wwwtax.cgi?id=265311) | 0.79 Mb | 12 | Mfl |
| Firmicutes | Mollicutes | [Mycoplasma hyopneumoniae 232](http://cmr.tigr.org/tigr-scripts/CMR/GenomePage.cgi?org=ntmh02) | [295358](http://www.ncbi.nlm.nih.gov/Taxonomy/Browser/wwwtax.cgi?id=295358) | 0.89 Mb | 3 | Mhy |
| Firmicutes | Mollicutes | [Mycoplasma mobile 163K](http://cmr.tigr.org/tigr-scripts/CMR/GenomePage.cgi?org=ntmm05) | [267748](http://www.ncbi.nlm.nih.gov/Taxonomy/Browser/wwwtax.cgi?id=267748) | 0.77 Mb | 5 | Mmo |
| Firmicutes | Mollicutes | [Mycoplasma mycoides SC PG1](http://cmr.tigr.org/tigr-scripts/CMR/GenomePage.cgi?org=ntmm02) | [272632](http://www.ncbi.nlm.nih.gov/Taxonomy/Browser/wwwtax.cgi?id=272632) | 1.21 Mb | 7 | Mmy |
| Firmicutes | Mollicutes | [Mycoplasma gallisepticum strain R](http://cmr.tigr.org/tigr-scripts/CMR/GenomePage.cgi?org=ntmg01) | [233150](http://www.ncbi.nlm.nih.gov/Taxonomy/Browser/wwwtax.cgi?id=233150) | 0.99 Mb | 3 | Mga |
| Firmicutes | Mollicutes | [Mycoplasma genitalium G-37](http://cmr.tigr.org/tigr-scripts/CMR/GenomePage.cgi?org=gmg) | [243273](http://www.ncbi.nlm.nih.gov/Taxonomy/Browser/wwwtax.cgi?id=243273) | 0.58 Mb | 2 | Mge |
| Firmicutes | Mollicutes | [Mycoplasma penetrans HF-2](http://cmr.tigr.org/tigr-scripts/CMR/GenomePage.cgi?org=ntmp03) | [272633](http://www.ncbi.nlm.nih.gov/Taxonomy/Browser/wwwtax.cgi?id=272633) | 1.35 Mb | 9 | Mpe |
| Firmicutes | Mollicutes | [Mycoplasma pneumoniae M129](http://cmr.tigr.org/tigr-scripts/CMR/GenomePage.cgi?org=ntmp01) | [272634](http://www.ncbi.nlm.nih.gov/Taxonomy/Browser/wwwtax.cgi?id=272634) | 0.81 Mb | 2 | Mpn |
| Firmicutes | Mollicutes | [Mycoplasma pulmonis UAB CTIP](http://cmr.tigr.org/tigr-scripts/CMR/GenomePage.cgi?org=ntmp02) | [272635](http://www.ncbi.nlm.nih.gov/Taxonomy/Browser/wwwtax.cgi?id=272635) | 0.96 Mb | 3 | Mpu |
| Firmicutes | Mollicutes | [Ureaplasma urealyticum parvum biovar serovar 3](http://cmr.tigr.org/tigr-scripts/CMR/GenomePage.cgi?org=ntuu01) | [95667](http://www.ncbi.nlm.nih.gov/Taxonomy/Browser/wwwtax.cgi?id=95667) | 0.75 Mb | 3 | Uur |
